# The Effects of Microsatellite Selection on Linked Sequence Diversity

**DOI:** 10.1093/gbe/evu134

**Published:** 2014-06-19

**Authors:** Ryan J. Haasl, Ross C. Johnson, Bret A. Payseur

**Affiliations:** ^1^Laboratory of Genetics, University of Wisconsin – Madison; ^2^Department of Biology, University of Wisconsin – Platteville; ^3^Present address: Department of Biology, University of Wisconsin – Platteville, Platteville, WI

**Keywords:** microsatellites, natural selection, genomic scans for selection, mutation, short tandem repeats

## Abstract

The genome-wide scan for selection is an important method for identifying loci involved in adaptive evolution. However, theory that underlies standard scans for selection assumes a simple mutation model. In particular, recurrent mutation of the selective target is not considered. Although this assumption is reasonable for single-nucleotide variants (SNVs), a microsatellite targeted by selection will reliably violate this assumption due to high mutation rate. Moreover, the mutation rate of microsatellites is generally high enough to ensure that recurrent mutation is pervasive rather than occasional. It is therefore unclear if positive selection targeting microsatellites can be detected using standard scanning statistics. Examples of functional variation at microsatellites underscore the significance of understanding the genomic effects of microsatellite selection. Here, we investigate the joint effects of selection and complex mutation on linked sequence diversity, comparing simulations of microsatellite selection and SNV-based selective sweeps. We find that selection on microsatellites is generally difficult to detect using popular summaries of the site frequency spectrum, and, under certain conditions, using popular methods such as the integrated haplotype statistic and SweepFinder. However, comparisons of the number of haplotypes (K) and segregating sites (S) often provide considerable power to detect selection on microsatellites. We apply this knowledge to a scan of autosomes in the human CEU population (CEPH population sampled from Utah). In addition to the most commonly reported targets of selection in European populations, we identify numerous novel genomic regions that bear highly anomalous haplotype configurations. Using one of these regions—intron 1 of *MAGI2*—as an example, we show that the anomalous configuration is coincident with a perfect CA repeat of length 22. We conclude that standard genome-wide scans will commonly fail to detect mutationally complex targets of selection but that comparisons of K and S will, in many cases, facilitate their identification.

## Introduction

The genome-wide scan for selection is a powerful method in the toolkit of the evolutionary biologist. Results from scans for selection can provide remarkable knowledge: The regions of the genome that have been among the most critical to the evolution of a population or species. For this reason and because whole-genome sequencing is becoming increasingly inexpensive, the genome-wide scan for selection first envisioned 40 years ago (Lewontin and Krakauer 1973) has now become commonplace (Biswas and Akey 2006; Akey 2009; Oleksyk et al. 2010; Strasburg et al. 2012). Moreover, scans for selection have lived up to their promise by identifying interesting examples of selection in a variety of species, including parallel evolution in divergent freshwater populations of threespine stickleback (Hohenlohe et al. 2010), local positive selection for a derived allele in the pigmentation gene *SLC24A5* in Europeans (Lamason et al. 2005), and selection for targeting ion transport and metal detoxification genes in the populations of *Arabidopsis lyrata* growing in inhospitable serpentine soils (Turner et al. 2008, 2010). As access to genomic data for an increasingly broad swath of phylogenetic diversity accrues, it becomes increasingly relevant to understand patterns of genome-wide polymorphism in as complete a way as possible. In particular, are there targets of selection that are overlooked by the scan for selection as currently practiced?

One particularly appealing feature of the genome-wide scan for selection is its ostensibly unbiased nature. Abstaining from a priori specification of candidate targets of selection, the genome-wide scan interrogates the majority of genomic regions without reference to their potential biological function—although a posteriori interpretation and follow-up experimentation may lead to bias and false conclusions (Thornton and Jensen 2007; Pavlidis, Jensen, et al. 2012). Although attention to potential ascertainment biases introduced by the researcher must be considered (Thornton and Jensen 2007), absent alternative explanations such as demographic change anomalous patterns of polymorphism may be cautiously attributed to natural selection. Yet, it is now clear that the models and statistics underlying genome-wide scans for selection may in fact lead to biased result sets with appreciable frequency. For example, selection from standing variation often fails to significantly distort patterns of genetic variation as measured by the site frequency spectrum (SFS; Innan and Kim 2005; Przeworski et al. 2005). Thus, standard genome-wide scans are biased toward identifying selective targets derived from new mutation. Similarly, selection on a polygenic trait may fail to significantly distort patterns of genetic variation linked to any one component gene (Pritchard et al. 2010; Pavlidis, Metzler, et al. 2012). Therefore, genome-wide scans may also be plagued by a bias toward the identification of genetic variants responsible for variation in Mendelian traits. Finally, Teshima et al. (2006) found that selective sweeps are more difficult to identify when the selected allele is recessive and concluded that this will lead genome-wide scans to produce an unrepresentative set of potential selective targets.

These and other biases associated with scans for selection have received substantial attention (Hermisson and Pennings 2005; Hancock, Alkorta-Aranburu, et al. 2010; Hancock et al. 2010). Here, we investigate a bias that is seldom considered. Namely, the methods of population genetics used to detect selection assume that positively selected variants emerge according to the infinite sites model (ISM; Kimura 1969). In other words, on the time scale of a selective event, the beneficial single-nucleotide variant (SNV) arises only once. Violations of the ISM in the context of sweeps targeting SNVs have been investigated—for example, infrequent recurrent mutation without back mutation (Pennings and Hermisson 2006a, 2006b). However, genomes are mutationally complex and functional variants are not limited to SNVs. For example, microsatellites are abundant in genomes and possess mutational rates and processes that are notably different from point mutation (Ellegren 2004). Selection targeting a microsatellite may affect linked sequence diversity in a fundamentally different manner than posited by the canonical model of selective sweeps (Maynard Smith 1976), meaning that standard genomic scans will fail to detect these targets of selection. In particular, the high mutation rate of microsatellites ensures that recurrent mutation is not an occasional event, as has been modeled by Pennings and Hermisson (2006a, 2006b) in the context of SNV-based selection.

Microsatellites are sequential repeats of a 1–6 nucleotide motif and their mutation does not follow the ISM (Ohta and Kimura 1973; Levinson and Gutman 1987; Weber and Wong 1993). Microsatellite mutation increases or decreases the number of repeats and occurs at a rate exceeding that of point mutation by several orders of magnitude (Bhargava and Fuentes 2010). This high mutation rate leads to recurrent mutation, back mutation, and multiallelism at microsatellite loci (Ellegren 2004).

Long considered to be nonfunctional genetic variants, a growing body of evidence suggests that a subset of microsatellites is functional. Numerous studies have identified a correlation between microsatellite variation at genic microsatellites and levels of gene expression (Rockman and Wray 2002; Vinces et al. 2009; Gemayel et al. 2010). In pathogenic bacteria, mutation of microsatellites found in open reading frames or their promoters cause phase variation by which phenotypes are turned on and off (Weiser et al. 1989; Moxon et al. 1994). Other microsatellites have been implicated in circadian clock regulation (Michael et al. 2007), drought tolerance in barley (Nevo et al. 2005), and skeletal morphology in domestic dog breeds (Fondon and Garner 2004). Microsatellite variation is often deleterious as well. For example, expansions of genic microsatellites cause a number of human neurological diseases (Orr and Zoghbi 2007) as well as canine epilepsy (Lohi et al. 2005). These diverse functional roles suggest that microsatellites may be targets of positive and negative natural selection.

The selective regime of a multiallelic microsatellite is necessarily more complex than that of a diallelic SNV. In conjunction with its complicated mutational properties, a microsatellite therefore represents a substantially different selective target than an SNV. Recently, we developed biologically realistic models of the diploid fitness surface at a nonneutral microsatellite (Haasl and Payseur 2013). These models were inspired by empirically observed correlations between microsatellite allele size (the number of times the motif is repeated) and gene expression (see Elmore et al. 2012 for an experimental investigation of the functions that relate allele size and gene expression in *Aspergillus flavus*). In most studied examples, the plot of gene expression versus allele size is a concave (Peters et al. 1999) or convex (Vinces et al. 2009) bell-shaped curve or a step-like graph in which expression increases or decreases suddenly at a threshold allele size (Okladnova et al. 1998; Yamada et al. 2000). In other words, the function relating allele size to gene expression is most readily divided into smooth and discontinuous cases. It therefore seems reasonable to model the genotypic fitness surface of a nonneutral microsatellite as either 1) a hill-like function in which one genotype is optimal with a relative fitness of 1 at the “top” of the hill (the additive and multiplicative models of Haasl and Payseur 2013) or 2) a surface that contains sharp divisions between high- and low-fitness genotypes (the dominant and recessive models of Haasl and Payseur 2013).

In this study, we investigate the selective footprint of microsatellite selection on linked variation for the first time. Using simulations, we vary mutation rate and selective strength, conduct comparisons with multiple scenarios of selection on SNVs, and examine the evolution of selective footprints through time. We compare the statistical power of several statistics that summarize sequence data to identify instances of microsatellite and SNV selection. We also examine the behaviors of the popular SweepFinder method (Nielsen et al. 2005) and integrated haplotype statistic (iHS; Voight et al. 2006) in response to microsatellite selection. We find that summaries of the SFS provide comparatively low power to detect selection at microsatellites, particularly when mutation rate is high. However, summaries of the haplotype distribution offer moderate-to-high power to detect selection on microsatellites. In particular, when conditioned on the number of segregating sites, the number of haplotypes provides considerable power to detect selection targeting highly mutable microsatellites. Finally, we use this knowledge to develop a test statistic sensitive to microsatellite selection, which we then apply in an illustrative scan for microsatellite selection in the CEPH population sampled from Utah (CEU).

## Materials and Methods

### Models of Selection and Mutation

#### Microsatellites

Throughout the article, we focus on two parameters that are useful for characterizing different instances of microsatellite selection. The first is the gradient parameter *g*, which controls the strength of selection. To see this, let *a_i_* represent a microsatellite allele with *i* repeats of a nucleotide motif (we refer to this as allele size *i*). Furthermore, assign the greatest relative fitness to an optimal allele size, *x*: $w(a_{x})=1$. Then, gradient parameter *g* determines the linear decline in fitness as distance from *x* increases and the relative fitness of each allele is then defined as $w(a_{i})=1-g|x-i|$. For example, if g=−0.01 and *x* = 10, then alleles of sizes 9 and 11 each have a relative fitness of 0.99. A stronger selective event, where g=−0.05, would assign relative fitnesses of 0.95 to allele sizes 9 and 11. Finally, the relative fitness of genotype $a_{i}a_{j}$ was calculated as $w(a_{i}a_{j})=[w(a_{i})+w(a_{j})]/2$. This is a simplified instance of the additive model presented in Haasl and Payseur (2013).

The second parameter used to characterize instances of microsatellite selection was the mutation parameter *ϕ*. We used a logistic model of microsatellite mutation rate, in which mutation rate is low for small allele sizes, increases dramatically at an intermediate allele size, and remains high for large allele sizes (supplementary fig. S1, Supplementary Material online). *ϕ* controls the maximum mutation rate at a locus. Each increase of *ϕ* by 1 increases maximum mutation rate by an order of magnitude. For example, although maximum mutation rate is $1\times 10^{-5}$ when ϕ=3, maximum mutation rate is $1\times 10^{-4}$ when ϕ=4. Mutation was symmetric, equally likely to increase or decrease allele size. Mutational step size followed a geometric distribution with *p* = 0.95, that is, 95% of mutations were single step.

#### SNVs

For comparison with microsatellite selection, we considered a diallelic SNV where the relative fitness of allele B was greater than that of allele b. To model positive selection at the locus, we used an additive selective regime in which relative genotypic fitnesses were w(BB)=1, w(Bb)=1−hs, and w(bb)=1−s. We set dominance coefficient *h* = 0.5 and selection coefficient *s* to either 0.05 or 0.01. We assumed a constant per-site point mutation rate of $2.5\times 10^{-8}$ and mutation followed the ISM (Kimura 1969).

### Simulation

We performed exact, forward-in-time simulations programmed in C++ and assumed a constant population size of $N_{\text{e}}=10,000$ (20,000 chromosomes). We varied the following parameters: *s* = 0.05 (strong SNV selection) or 0.01 (weak SNV selection); ϕ=3 (low microsatellite mutational pressure) or 5 (high microsatellite mutational pressure); and g=−0.01 (weak microsatellite selection) or −0.05 (strong microsatellite selection). For each distinct combination of parameter values, we ran 500 simulation replicates. In the case of SNV selection, we noted the generation at which the beneficial SNV became fixed in the population. In simulations of microsatellite selection, we noted the equilibrium generation, which we defined as the first generation for which the difference between the frequency of the most fit allele *a_x_* and its frequency at mutation–selection balance (determined in the absence of genetic drift; Haasl and Payseur 2013) was less than $1/2N=5\times 10^{-5}$. Most simulated sequences were 1 Mb in length, although we also simulated 30-kb sequences for efficiency in some cases. All simulations assumed a recombination rate of ρ=1.25 cM/Mb.

#### Neutral, Preselection Phase

For each simulation replicate, we used neutral coalescent simulations implemented in MS (Hudson 2002) to obtain a starting population of 20,000 chromosomes (*N_e_* = 10,000 diploids). We then extracted the genealogy corresponding to the exact center of the simulated 1-Mb or 30-kb sequence. In the case of microsatellite selection, we input this genealogy to the program MARKSIM (supplementary text, Supplementary Material online; Haasl and Payseur 2011), which outputs a starting microsatellite allele for each chromosome. In all cases, we specified allele size of 8 as the MRCA of the genealogy. The only significance of this allele size was that it was sufficiently large to provide modest mutability at the locus, which more often than not resulted in a microsatellite locus that entered the selective phase as polymorphic. The microsatellite locus was placed at the exact center of the simulated sequence and the allele size of the most fit allele was determined randomly in the interval [8, 20]. Thus, for many replicates the most fit allele did not exist in the population when selection began. For simulations of SNV-based sweeps from standing variation, we also used the genealogy corresponding to the center of the simulated sequence. We searched this tree for a bipartition that allowed us to generate a new SNV at the center of the sequence with a minor allele frequency in the interval [0.1, 0.15]. In rare cases where a suitable bipartition was unavailable, we simply started the simulation over. The minor allele was treated as the beneficial SNV. In simulations of a hard selective sweep, we simply placed a single copy of a beneficial SNV at the center of one random chromosome. All other chromosomes carried the less fit ancestral allele.

#### Selection Phase

The selective phase proceeded as follows:

1. Set generation counter to 1.
2. SELECTION: Determine which of the 10,000 individuals survive to reproduce based on the genotypic fitness of the selected SNV or microsatellite genotype.
3. REPRODUCTION and HOMOLOGOUS RECOMBINATION: Use the pool of survivors from step 1, and repeat the following steps until 10,000 offspring are generated:
  - Randomly choose two parent individuals
  - Determine if homologous recombination occurs; if so, perform crossover, yielding 2 recombinant and 2 nonrecombinant chromosomes
  - Choose one chromosome from each parent for inheritance by the offspring
4. MUTATION: For each chromosome of the next generation, randomly determine how many (if any) new SNVs arise (Poisson distributed) and at what position(s). Check for mutation at the microsatellite.
5. (SNV selection only) If the beneficial SNV is lost, set generation counter to 1 and start selective phase over from the original set of starting chromosomes.
6. Determine if fixation (SNVs) or mutation–selection balance (microsatellites) has been achieved. Increment generation counter and return to step 2.

We stopped simulations of 1-Mb sequence at the point of fixation/equilibrium. For simulations of 30-kb sequence, we simulated 2,000 additional generations beyond the point of fixation/equilibrium following. In the case of SNV selection, postfixation generations did not require performance of step 2.

#### Sampling

At each sampling time point, we randomly sampled 50 individuals (100 chromosomes) from the population. For 1-Mb simulations, we only sampled the population upon fixation/equilibrium. For simulations of 30-kb sequence, we sampled every generation prior to fixation/equilibrium and then at the following time points: Fixation/equilibrium and 100, 250, 500, 1,000, and 2,000 generations afterward.

#### Measuring the Distance between Starting and Equilibrium Allele Frequencies at a Microsatellite Targeted by Selection

For a microsatellite under selection, we previously showed that the duration and cost of selection (i.e., death due to selection against suboptimal genotypes) are positively correlated with the distance between the starting allele frequencies and those at mutation–selection equilibrium (Haasl and Payseur 2013). Because the most fit allele size and the starting distribution of allele sizes were randomly determined for each replicate, this distance varied between replicates. We quantified this consequential distance as

$$\Delta_{\text{msat}}=\sum_{x\in S}^{}\sum_{y\in E}^{}|x-y|p_{x}p_{y},$$

where S is the set of starting allele sizes, E is the set of equilibrium allele sizes, and $p_{•}$ is the allele frequency. The equilibrium alleles of set E and their frequencies *p_y_* were determined using a single deterministic simulation for the appropriate selective and mutational parameter values.

#### Nonequilibrium Demography

Because changes in population size can substantially alter patterns of genetic variation—often in ways that mimic selective events—it is important to investigate the effect of demographic change on our ability to detect selection. We modeled two common demographic scenarios: 1) Bottleneck expansion (a population bottleneck followed by an exponential population expansion) and 2) exponential decline. In both cases, the onset of demographic change coincided with the onset of selection. We modeled an instantaneous bottleneck that reduced population size from 10,000 diploids to 500 diploids. The subsequent expansion was exponential with a per-generation rate-of-increase of 0.005. In the case of exponential population decline, we used a per-generation rate-of-decrease of −0.003. We simulated hard sweeps, microsatellite selection (g=−0.05;ϕ=5), and neutral evolution under both scenarios. Samples of 100 chromosomes were drawn when the beneficial allele fixed (SNV) or mutation–selection balance was achieved (microsatellite). By comparing the simulations of neutral evolution under these demographic scenarios with those of SNV and microsatellite selection under the same demographic scenarios, we modeled the real-world situation in which a researcher generates a null distribution using an accurate estimate of the focal population’s demography.

### Summary Statistics

We calculated the following statistics for all simulations: 1) Tajima’s *D* (Tajima 1989); 2) Fay and Wu’s *H*_FW_ (Fay and Wu 2000); 3) Zeng et al.’s *E* (Zeng et al. 2006); 4) number of distinct haplotypes *K*; 5) haplotype diversity *H*; and 6) count of the most frequent haplotype *M*. The first three statistics are separate estimators of the scaled mutation rate $\theta =4N_{\text{e}}\mu$, where *N*_e_ is the effective population size and μ the mutation rate. Although these estimators possess identical expectations at mutation–drift equilibrium, they diverge from each other in characteristic ways under nonequilibrium conditions due to dependencies on different partitions of the frequency spectrum (Zeng et al. 2006). The final three statistics summarize the distribution of sampled haplotypes. Each statistic was separately calculated for each nonoverlapping 10-kb window in the simulated sample of 1-Mb or 30-kb sequences.

Finally, we defined a seventh summary statistic meant to capture the large differences between *K* and *S* observed in simulations of microsatellite selection:

$$ksk_{\left(n\right)}^{2}=n^{-1}\sum_{i=1}^{n}\frac{K_{i}-S_{i}}{K_{i}^{2}}$$

where *n* is the number of contiguous windows, and *K_i_* and *S_i_* are the number of unique haplotypes and segregating sites observed in the *i*th of *n* windows, respectively. We divide $K_{i}-S_{i}$ by $K_{i}^{2}$ because our simulations indicated that a defining signature of microsatellite selection was a substantial decrease in *K* accompanied by a more modest decline in *S*. Thus, a large value of $K_{i}-S_{i}$ is more likely to signal microsatellite selection when *K* is small; dividing the difference by *K*^2^ inflates the magnitude of the statistic when *K* is small. Another motivation for using this statistic is that it does not require the computation of separate empirical distributions for each value of *S* as the test statistic K|S does (Innan et al. 2005). Although *n* could be any value, we use *n* = 20 (10-kb windows) in a scan for selection (see below). Stepping across a sequence one window at a time, $ksk_{\left(20\right)}^{2}$, then provides a moving average that indicates broad, 200-kb regions where the disparity between *K* and *S* is pronounced.

### Power Analyses

Scaled mutation and recombination parameters, θ and ρ, respectively, can vary widely across the genome. Unfortunately, equilibrium values of the statistics we measured here depend on the values of these two parameters. To incorporate empirical uncertainty regarding θ and ρ, we computed empirical null distributions for each statistic based on $10^{6}$ neutral coalescent simulations of 10-kb sequences (*n* = 100) in MS (Hudson 2002), which each began with independent draws from uniform prior probability densities for θ and ρ. We considered reasonable ranges of these parameters for human: Recombination rates between 0.75 and 2.0 cM/Mb, per-site point mutation rate μ between $5\times 10^{-9}$ and $2.5\times 10^{-8}$, and effective population size *N_e_* between 10,000 and 25,000. For a 10-kb sequence, these imply priors of θ∼ [2, 25] and ρ∼ [3, 20]. The empirical distribution for each statistic was conditioned on the number of segregating sites, *S*, and was simply the distribution of the statistic across the subset of simulated 10-kb windows in which *S* = *s*.

We calculated power using the results from 30-kb simulations, in which the selective target was positioned at the midpoint of the 30-kb sequence. For each statistic, we tested each of the three nonoverlapping 10-kb windows for significance and counted selection as detected if one or more of the three windows produced a significant result. The positive selection modeled here is expected to shift each statistic in one specific direction. Therefore, all tests were one tailed. Values of statistics such as Tajima’s *D*, which is expected to decrease in response to positive selection, were deemed significant if they ranked below the α=0.05/3=0.0167 quantile of the appropriate empirical distribution. *M*, on the other hand, is expected to increase in response to positive selection and was deemed significant if its rank was greater than or equal to the 1−0.05/3=0.9833 quantile of the appropriate empirical distribution. We calculated the power of a statistic as the fraction of 500 replicates in which selection was detected by the statistic.

### SweepFinder and iHS

To examine the behaviors of the SweepFinder method (Nielsen et al. 2005) and iHS (Voight et al. 2006) in response to microsatellite and SNV-based selection, we simulated 60 unlinked 1-Mb sequences. Forty of the 1-Mb sequences were simulated under neutral conditions and 20 were the targets of either microsatellite selection or an SNV-based hard sweep. Because the iHS has been shown to have maximal power before the selected SNV reaches fixation (Voight et al. 2006), we included simulations of selection where the selected SNV or microsatellite was 1) at 60% of fixation or equilibrium and 2) at fixation or equilibrium. By chance, the selected microsatellites had a wide range of $\Delta_{\text{msat}}$ values.

We calculated the overall frequency spectrum for all 60 loci using SweepFinder (grid size was set such that one value of the composite likelihood ratio was calculated every 10 kb) and assumed this frequency spectrum in the individual analyses of each locus. We used the R package *rehh* (Gautier and Vitalis 2012) to calculate integrated haplotype homozygosity (iHH) for each of the 60 loci, concatenated these results, and used *rehh* to calculate standardized iHH (iHS). When calculating iHH, we excluded SNVs with minimum allele frequencies <0.05.

### Scan for Human Microsatellite Selection

From the 1000 Genomes project Web site (1000 Genomes Project Consortium 2010), we downloaded variant call files for all autosomes from 85 individuals (*n* = 170) in the CEU population (CEPH individuals sampled from Utah and with northern and western European genetic ancestry). Genotypes were phased using BEAGLE (Browning SR and Browning BL 2007) or MACH (Li et al. 2010). We divided each chromosome into nonoverlapping 10-kb windows and calculated $ksk_{\left(20\right)}^{2}$ for each set of 20 contiguous windows along each chromosome. We use 10-kb windows because this is the resolution of recent estimates of human recombination rate (Kong et al. 2010). We average over 20 windows because this provides a smooth plot relative to $ksk_{\left(1\right)}^{2}$ plots, which allows easy identification of anomalous regions. The position of each value of $ksk_{\left(20\right)}^{2}$ was associated with its midpoint.

To assess the significance of observed values of $ksk_{\left(20\right)}^{2}$, we performed 180,000 coalescent simulations of 2-Mb sequences using MS (Hudson 2002). We assumed a uniform prior on per-site recombination rate of $1\times 10^{-9}$ through $1\times 10^{-8}$ and based priors of demographic parameters on the estimates of Gravel et al. (2011). See supplementary material, Supplementary Material online, for commands and prior distributions on other parameters of importance. For each simulated 2-Mb sequence, we then calculated $ksk_{\left(20\right)}^{2}$ for each of its 181 distinct 20-window sequences. This approach to the simulation of $ksk_{\left(20\right)}^{2}$ values accounts for the autocorrelation between the component windows in a 20-window stretch. The empirical null distribution included 32.58 million values of $ksk_{\left(20\right)}^{2}$. To correct for multiple tests, we used a false discovery rate (FDR) threshold of 2%.

## Results

### The Spatial Footprint of Selection on Microsatellites

#### SFS-Based Statistics

**Fig. 1.—:**
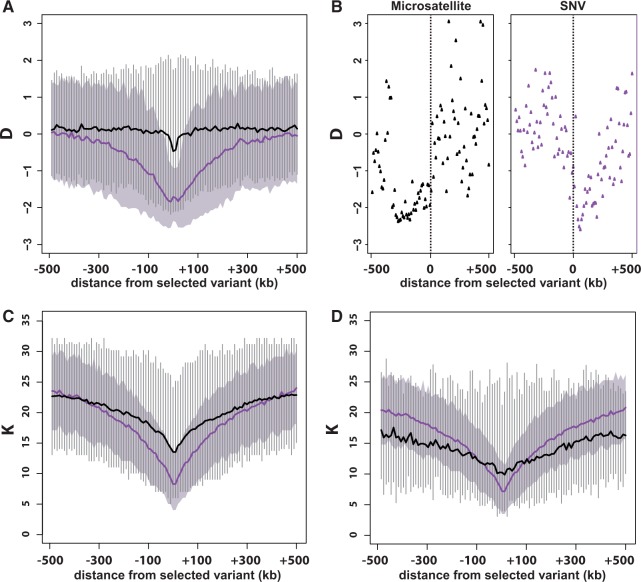
The spatial footprint of a hard sweep compared with that of selection on a microsatellite. (*A*) Tajima’s *D* summarized across 500 simulations of a hard sweep (s=0.05,h=0.5) or selection on a microsatellite (additive model, ϕ=5, g=−0.05). *D* was measured in the generation following fixation of the beneficial SNV (hard sweep) or achievement of mutation–selection equilibrium (microsatellite selection). Purple and black lines mark the mean value of *D* across 500 simulations of a hard sweep and microsatellite selection, respectively. The 5–95% interquantile range of *D* is marked by a light purple cloud (hard sweep) or vertical gray bars (microsatellite selection). (*B*) Results from a single simulation of microsatellite selection (left) and a hard sweep targeting an SNV (right). Points mark the value of *D* at each nonoverlapping 10-kb window across the simulated 1-Mb sequence. Vertical dashed line indicates the position of the selected SNV or microsatellite. (*C*) The number of haplotypes *K*. Colors are the same as in (*A–B*). (*D*) Same as (*C*), except only microsatellites with values of $\Delta_{\text{msat}}$ in the top 10% of all simulations are included.

On average, SFS-based statistics were more sensitive to a hard sweep than selection on microsatellites. The spatial footprint of selection as measured by Tajima’s *D* (Tajima 1989) is shown in figure 1*A*. For SNV-based selection, these measures were taken immediately after fixation of the favored SNV. For microsatellite selection, they were taken on achievement of mutation–selection equilibrium at a selected microsatellite. In the case of microsatellite selection, the mean value of *D* was flat around zero (black line) except for a minor deflection at the position of the targeted microsatellite. This result contrasts sharply with the deep trough in mean *D* seen in simulations of a hard sweep on an SNV (purple line).

However, *D* showed considerable variance across simulation replicates. Figure 1*B* (right panel) shows the values of *D* from one simulation of a hard sweep. In keeping with previous results (Kim and Stephan 2002), downward deflection in *D* was often asymmetrical relative to the selected SNV. In the case of microsatellites, some simulation replicates demonstrated dramatic departures from the mean value of *D* for microsatellites. Figure 1*B* (left panel) shows an illustrative microsatellite simulation in which Tajima’s *D* was primarily deflected downward to the left of the selected microsatellite. Although this is qualitatively similar to the SNV (hard sweep) case, the width of the trough in *D* values is much wider. In addition, this replicate of microsatellite selection affected linked variation at a much longer range than in the hard sweep case, with values of D<−2 in excess of 300 kb from the selected microsatellite. Also of note, in this same simulation replicate, we observed highly positive values of *D* to the right of the selected microsatellite, which illustrates the comparatively higher variance in *D* and other summary statistics associated with microsatellite selection. Many simulations of microsatellite selection that used parameter values identical to those illustrated in figure 1*B* (except for starting allele frequency distribution and the favored allele size, which were drawn randomly) only generated moderately positive and/or negative values of *D* across the entire simulated 1-Mb sequence. Thus, microsatellite selection produced a highly variable and often very weak effect on the values of SFS-based statistics such as *D*. However, when *D* was driven negative by microsatellite selection, the decreases were often substantial, expansive, and long ranged. Spatial patterns of Fay and Wu’s *H*_FW_ (Fay and Wu 2000) and Zeng et al.’s *E* (Zeng et al. 2006) were qualitatively similar to those observed in *D* (supplementary fig. S2, Supplementary Material online).

#### Haplotype-Based Statistics

The average decline in *K* (the number of unique haplotypes) was similar whether the target of selection was an SNV or microsatellite. However, limiting consideration of microsatellite selection to the 10% of simulations with the highest values of $\Delta_{\text{msat}}$—which quantifies the difference between allele frequencies at the start of selection and mutation–selection equilibrium—we observed a much broader selective footprint in the case of microsatellite selection (fig. 1*D*). As with SFS-based statistics, microsatellite selection resulted in greater inter- and intrareplicate variability in haplotype-based statistics. This fact is evident in the much broader interquantile (5–95%) ranges of *K* for simulated microsatellite selection (fig. 1*C* and *D*).

### The Temporal Footprint of Selection on Microsatellites

#### SFS-Based Statistics

**Fig. 2.—:**
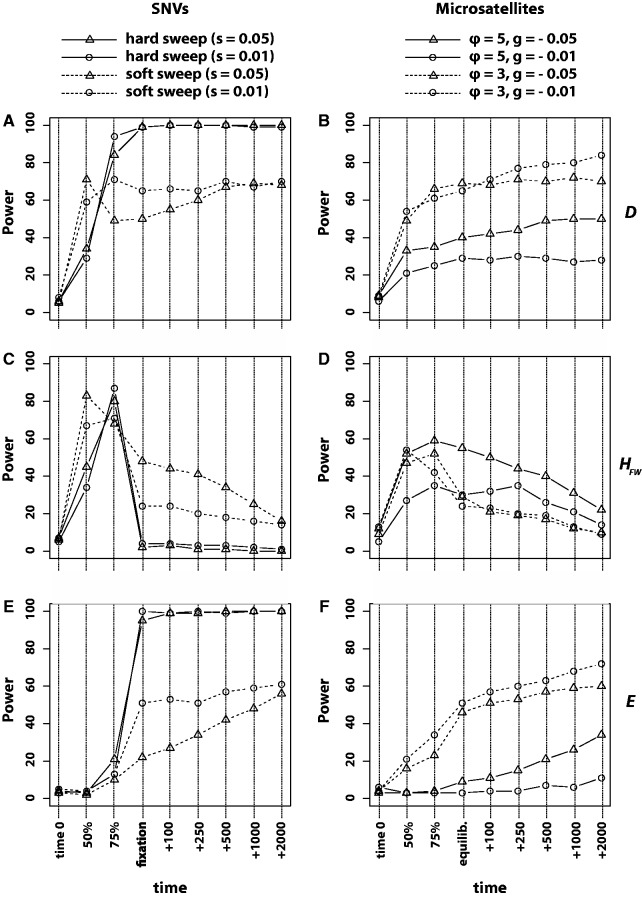
Statistical power of statistics that summarize the site frequency spectrum. Power to detect sweeps targeting SNVs is shown in the left column, whereas power to detect scenarios of microsatellite selection is shown in the right column. (*A*, *B*) The power of Tajima’s *D*. (*C*, *D*) The power of Fay and Wu’s *H*_FW_. (*E*, *F*) The power of Zeng et al.’s *E*. Time points sampled are as follows: Time 0, the generation before selection begins; 50%, half the time to fixation/equilibrium; 75%, three-quarters the time to fixation/equilibrium; fixation/equilibrium, one generation after fixation or mutation–selection equilibrium; +*X*, *X* generations after fixation or mutation–selection equilibrium.

The power of SFS-based statistics to detect selection varied considerably over time and by selective target (fig. 2). For selection targeting SNVs (hard and soft sweeps), *D* increased to high statistical power by the time of fixation of the favored SNV. The power afforded by *D* was consistent to the last time point sampled (2,000 generations = 0.05 $4N_{\text{e}}$ generations postfixation). On the other hand, the power of *H*_FW_ declined precipitously following fixation of the favored SNV, particularly in the case of a hard sweep (fig. 2*C*). Finally, *E* provided high power to detect selection, but only following fixation of the favored SNV (fig. 2*E*).

The power of these same statistics to detect microsatellite selection was comparatively muted. *D* and *E* showed increasingly high power to detect selection after mutation–selection equilibrium was achieved, particularly when the mutation rate of the selected microsatellite was low (dashed lines, fig. 2*B* and *F*). However, when microsatellite mutation rate was high, the power of these two statistics to detect microsatellite selection was considerably less than their power to detect selection on SNVs (hard or soft sweeps). In particular, when ϕ=5 (high mutational pressure at the microsatellite), *E* only began to register selection hundreds of generations after mutation–selection equilibrium was achieved (solid lines, fig. 2*F*). *H*_FW_ maintained power to detect microsatellite selection after mutation–selection equilibrium, although power was low to moderate (fig. 2*D*).

#### Haplotype-Based Statistics

**Fig. 3.—:**
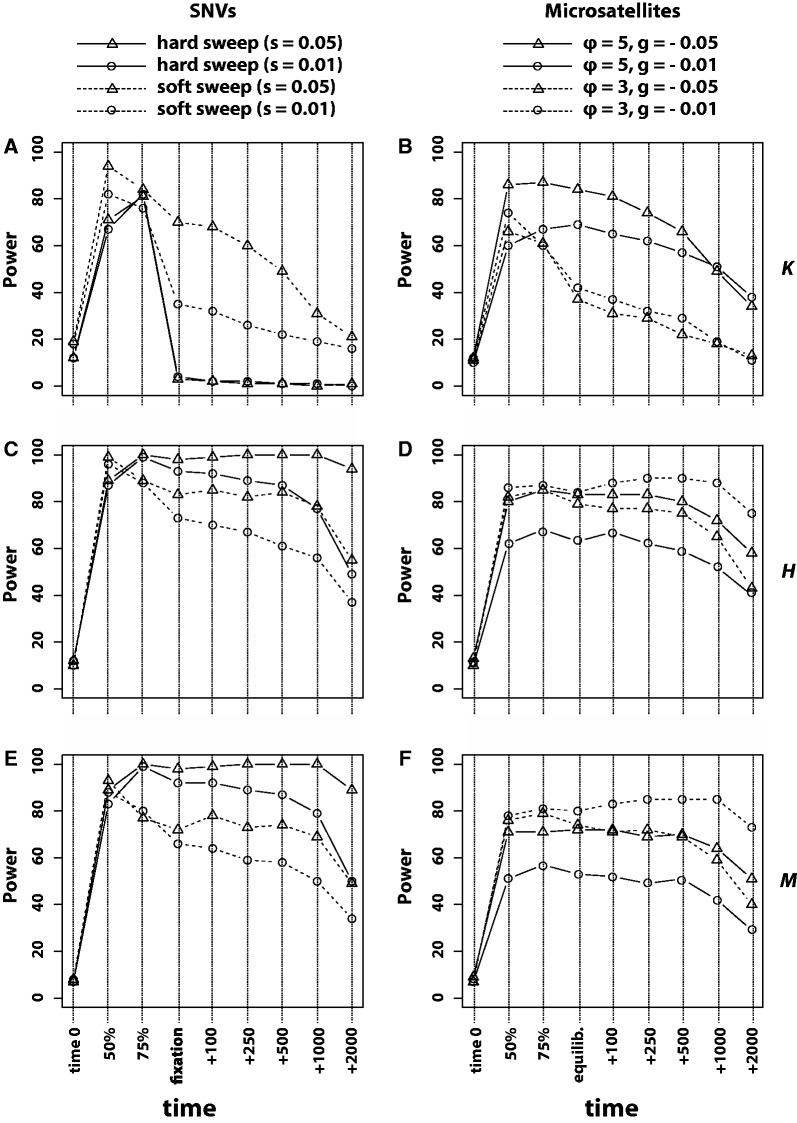
Statistical power of statistics that summarize the distribution of haplotypes. Power to detect sweeps targeting SNVs is shown in the left column, whereas power to detect scenarios to microsatellite selection is shown in the right column. (*A*, *B*) The power of *K*. (*C*, *D*) The power of *H*. (*E*, *F*) The power of *M*. Time points sampled are the same as in figure 2.

Both haplotype diversity, *H*, and frequency of the most frequent haplotype, *M*, maintained intermediate-to-high power to detect selection long after fixation in the case of positive selection targeting an SNV (fig. 3*C* and *E*). Conversely, the power of *K* declined rapidly following fixation of the beneficial SNV. In the case of SNV selection (hard sweep), the statistical power of *K* declined to near zero following fixation. On the other hand, *K* provided intermediate-to-high power to detect microsatellite selection before and after mutation–selection equilibrium was achieved (fig. 3*B*). Unlike other statistics, the power of *K* to detect microsatellite selection was markedly higher when mutation rate of the targeted microsatellite was high. Additionally, the power of *K* to detect selection on a highly mutable microsatellite was greater than the power of *K* to detect selection on SNVs (hard or soft sweeps). Both *H* and *M* demonstrated intermediate-to-high power to detect microsatellite selection, although lower power than for hard sweeps targeting SNVs (fig. 3*C*–*F*).

#### Haplotype Configuration and the Uniqueness of the Most Common Haplotype Relative to Other Haplotypes

**Fig. 4.—:**
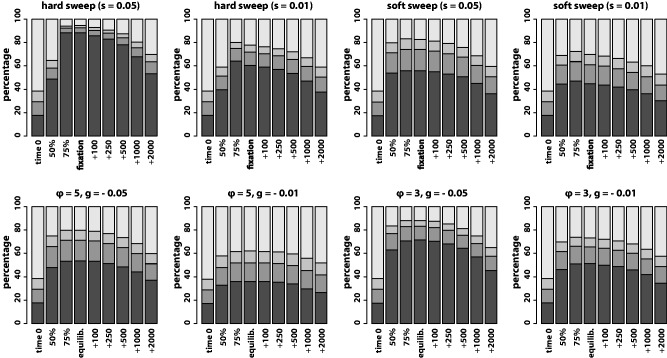
Changes in haplotype configuration through time. Each panel is labeled with the corresponding selective scenario and proportions illustrated are average proportions across 500 simulations each. The proportions of the sample of the first, second, and third most common haplotypes are shaded in decreasingly dark shades of gray. The proportion of the remaining haplotypes is shaded lightest. Time points sampled are the same as in figures 2 and 3.

Haplotype configuration differed markedly among selective scenarios and selective targets (fig. 4). As expected, a hard sweep and strong selection (*s* = 0.05) drove a single haplotype to near fixation, implying a drastic loss of diversity that facilitated comparatively easy detection of hard sweeps using SFS-based statistics (fig. 2*A* and *E*). Selection on microsatellites with high mutation rate (ϕ=5) produced haplotype configurations in which the three most common haplotypes all had frequencies greater than 10% on average and the most common haplotype was found at a frequency of ≤0.5. In other words, multiple haplotypes became common and remained so for hundreds to thousands of generations.

#### A Test Statistic for Selection on Highly Mutable Microsatellites

*K* declined markedly in cases of both SNV and microsatellite selection. However, when more than one haplotype remains following fixation of a beneficial SNV or achievement of mutation–selection balance, these haplotypes are likely to be less similar in the case of microsatellite selection. We expect this because beneficial microsatellite alleles may arise on multiple haplotypic backgrounds that are dissimilar, whereas hard sweeps drive a single haplotype to (or near) fixation. By definition, a set of divergent haplotypes collectively contains a greater number of segregating sites, *S*, than a set of similar haplotypes. Thus, although a variety of selective events lower *K* substantially, microsatellite selection may be somewhat unique in its simultaneous maintenance of segregating sites, *S*. To take advantage of the disparity between *K* and *S* observed in simulations of microsatellite selection, we proposed the test statistic $ksk_{\left(n\right)}^{2}$ (see Materials and Methods). This statistic assumes negative values whenever *S* > *K* and its absolute value increases as *K* declines. Thus, highly negative values of $ksk_{\left(n\right)}^{2}$ indicate that S>>K and that *K* is small.

We calculated the power of $ksk_{\left(20\right)}^{2}$ to detect selection on SNVs (*s* = 0.01 and *s* = 0.05) and microsatellites with different mutation rates (ϕ=3 and ϕ=5). We also considered three time points. For each combination of selective target and time point, we used 500 independent simulations and compared the values of $ksk_{\left(20\right)}^{2}$ with a null distribution derived from $1\times 10^{6}$ neutral simulations. The null distribution was simply the collection of the most extreme value of $ksk_{\left(20\right)}^{2}$ (among 100 10-kb windows) from each replicate. A selection replicate was considered significant if it produced a value of $ksk_{\left(20\right)}^{2}$ that was less than the critical value of −0.071 (Bonferroni corrected).

We found that $ksk_{20}^{2}$ possesses no power to detect hard sweeps of limited strength (*s* = 0.01) and high power to detect strong selective sweeps targeting an SNV (table 1). $ksk_{\left(20\right)}^{2}$ possesses intermediate to high power to detect microsatellite selection regardless of $\Delta_{\text{msat}}$ and across reasonable rates of mutation (ϕ=3 or ϕ=5). The statistic possesses long-lived power to detect microsatellite selection whenever $\Delta_{\text{msat}}$ is intermediate to high (>4; table 1). Together, these results show that $ksk_{\left(20\right)}^{2}$ can detect a variety of microsatellite selective targets for many generations following achievement of mutation–selection balance.

**Table 1:** Power of $ksk_{\left(20\right)}^{2}$ to Detect Selection on Various Targets at Fixation/Equilibrium and Beyond

Target of Selection	Power		
	Fixation/ Equilibrium	250 Generations Post	500 Generations Post
SNV (*s* = 0.01)	0.033	0.047	0.041
SNV (*s* = 0.05)	0.998	0.976	0.742
Microsatellite (ϕ=3; all msats)	0.802	0.649	0.483
Microsatellite (ϕ=5; all msats)	0.700	0.609	0.453
Microsatellite (ϕ=3; $\Delta_{\text{msat}}>4$)	0.969	0.900	0.645
Microsatellite (ϕ=5; $\Delta_{\text{msat}}>4$)	0.929	0.851	0.776

### Comparing the Behaviors of SweepFinder, iHS, and $ksk_{\left(20\right)}^{2}$

**Fig. 5.—:**
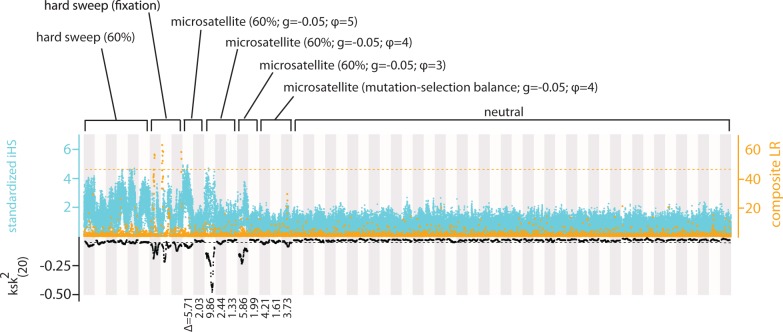
A comparison of $ksk_{\left(20\right)}^{2}$, iHS, and the composite likelihood ratio. We simulated 60 1-Mb sequences under neutral, partial hard sweep (*s* = 0.05), complete hard sweep (*s* = 0.05), and microsatellite selection scenarios. Simulated scenarios are indicated above the graph. $ksk_{\left(20\right)}^{2}$ values are in black, iHS values are in blue, and composite likelihood ratios are in orange. The dashed black line coincides with the lowest observed value of $ksk_{\left(20\right)}^{2}$ among the 40 neutral simulations. The dashed orange line is Bonferroni-corrected significance threshold for the composite likelihood ratio based on 1 million neutral simulations performed in SweepFinder.

For microsatellite selection, we varied the time at which the sample was taken (at mutation–selection equilibrium or 60% of equilibrium), as well as the values of the gradient parameter (−0.01 or −0.05) and mutation parameter *ϕ* (3, 4, or 5). By chance, the values of $\Delta_{\text{msat}}$ also varied (fig. 5). All instances of microsatellite selection failed to generate statistically significant values of the composite likelihood ratio. However, the iHS and $ksk_{\left(20\right)}^{2}$ statistics varied widely under the different conditions simulated. By far, the most important factor determining the magnitude of these statistics was $\Delta_{\text{msat}}$. All three instances where $\Delta_{\text{msat}}$ was >5 resulted in strongly positive values of iHS and strongly negative values of $ksk_{\left(20\right)}^{2}$ despite the fact that three different values of mutation parameter *ϕ* were used. Importantly, after mutation–selection balance was achieved, values of iHS no longer exceeded those commonly found in simulations of neutral evolution, whereas $ksk_{\left(20\right)}^{2}$ remained marginally significant (fig. 5).

Samples of genetic variation (*n* = 100 chromosomes) taken when a favored SNV achieved a frequency of 0.6 (fig. 5; hard sweep [60%]) revealed strong outlier values of iHS when compared with samples from simulations under neutral conditions. In some cases, these same samples showed values of $ksk_{\left(20\right)}^{2}$ that were slightly lower than the minimum value of $ksk_{\left(20\right)}^{2}$ achieved in neutral simulations (fig. 5; black, dashed line). None of these samples generated statistically significant values of the composite likelihood ratio calculated using SweepFinder.

Samples taken the generation after a favored SNV fixed (fig. 5; hard sweep [fixation]) showed elevated values of iHS relative to neutral samples, although less elevated than for partial sweeps. These were the only samples to produce significant values of the composite likelihood ratio in SweepFinder. Values of $ksk_{\left(20\right)}^{2}$ were highly negative for these samples; closer inspection revealed that values of $ksk_{\left(20\right)}^{2}$ were most negative in the regions flanking the favored SNV and nearly positive at the position of the favored SNV (fig. 5).

To summarize, partial hard sweeps produced strongly elevated values of iHS, middling deflections of $ksk_{\left(20\right)}^{2}$, and no significant values of the composite likelihood ratio. Completed hard sweeps produced strong deflections of all three statistics, including statistically significant values of the composite likelihood ratio. Finally, results varied widely in the case of microsatellite selection, but in cases where $\Delta_{\text{msat}}$ was large, both iHS and $ksk_{\left(20\right)}^{2}$ were strongly deflected. Only $ksk_{\left(20\right)}^{2}$ was significantly different from neutral expectations once mutation–selection balance was achieved in cases of microsatellite selection (fig. 5).

We also compared the behavior of these three methods in two cases of nonequilibrium demography: Bottleneck expansion and exponential decline. We used comparative neutral distributions that were simulated under the true demographic model. $ksk_{\left(20\right)}^{2}$ was well powered to detect selective events (SNV and microsatellite) under these two cases of demographic change (supplementary figs. S3–S6, Supplementary Material online). For a variety of $\Delta_{\text{msat}}$ values, $ksk_{\left(20\right)}^{2}$ values were markedly different from those produced under neutral conditions (supplementary figs. S3 and S4, Supplementary Material online). If we remove the assumption that a researcher will be able to accurately estimate the true demographic model (from which a useful null distribution can be simulated), our simulations suggest that outlier methods that simply identify the most extreme values of $ksk_{\left(20\right)}^{2}$ could be used effectively. In all cases of microsatellite selection (under both demographic models), a substantial trough of $ksk_{\left(20\right)}^{2}$ was observed that was noticeably lower than the background level (supplementary fig. S7, Supplementary Material online). In the case of microsatellite selection and exponential population decline, we observed outlier values of standardized iHS (supplementary fig. S3, Supplementary Material online). For both SNV and microsatellite selection and both demographic models, SweepFinder consistently identified high values of the composite likelihood ratio (LR) relative to neutral simulations. We note, however, that these high values are relatively sparse and do not consist of clear “towers” of contiguous significant windows as seen in simulations of constant population size (fig. 5).

Although iHS and SweepFinder represent two of the most popular statistics/methods for detecting selective sweeps, we note that there are several others we did not test here. Perhaps, the most promising statistic in the current context is ω (Kim and Nielsen 2004; Alachiotis et al. 2012), which compares relative linkage disequilibrium on either side of a focal point to identify selective targets.

### Scan for Microsatellite Selection

Because simulations indicated that highly negative values of $ksk_{\left(20\right)}^{2}$ are expected in sequences linked to highly mutable microsatellites experiencing selection (figs. 3–5), we scanned the human autosomes for extreme values of the proposed test statistic $ksk_{\left(20\right)}^{2}$ in a sample of autosomes (*n* = 170) from the CEU population. Comparing 262,575 values of $ksk_{\left(20\right)}^{2}$ from across the autosomes with an empirical null distribution indicated that values of $ksk_{\left(20\right)}^{2}<-0.073$ were significant (2% FDR). Three thousand two hundred twenty-eight values of $ksk_{\left(20\right)}^{2}$ (1.23% of all 10-kb windows) surpassed this significance threshold. However, this set comprised 233 clusters of extreme $ksk_{\left(20\right)}^{2}$ values at distinct genomic locations (supplementary table S1, Supplementary Material online).

Nearly all of the most commonly reported targets of selection in European populations were found in or within 1 Mb of one of the 233 clusters of extreme $ksk_{\left(20\right)}^{2}$ values: *LCT*, intergenic region 4p15.1, *FOXP2*, *SLC24A5*, *BCAS3*, *HERC2* (Voight et al. 2006; Sabeti et al. 2007; Sturm et al. 2008). However, of the 37 clusters with an extreme $ksk_{\left(20\right)}^{2}\leq -0.1$, 27 coincided with regions absent from any of the 9 high-profile genomic scans documented by Akey (2009) (table 2). Interestingly, 15 of 233 extreme $ksk_{\left(20\right)}^{2}$ clusters were coincident with clusters of olfactory receptor (chr3: 98,020,000; chr6: 29,380,000; chr7: 142,660,000; chr11: 55,770,000; chr11: 124,150,000), zinc finger (chr5: 150,280,000; chr9: 99,570,000; chr19: 22,840,000; chr19: 40,560,000), serine protease inhibitor (chr5: 147,530,000; chr18: 61,550,000), toll-like receptor (chr4: 38,800,000), major histocompatibility complex (chr6: 30,040,000), caspase (chr11: 104,720,000), or keratin-associated protein (chr21: 32,070,00) genes.

**Table 2:** Most Extreme Values of $ksk_{\left(20\right)}^{2}$ in Scan of CEU Genomes, *n* = 170 Chromosomes

Chromosome	Position	$ksk_{\left(20\right)}^{2}$	Genes	Overlap (Akey 2009)
4	171510000	−0.141	Intergenic	4 scans
8	12490000	−0.139	LOC100506990*, LOC729732*, FAM86B2,	
			*LONRF1, LOC340357*	None
4	148680000	−0.139	PRMT10*, TMEM184C, ARHGAP10	4 scans
18	58410000	−0.138	Intergenic	None
4	64610000	−0.134	Intergenic	None
6	29380000	−0.125	OR5V1*, OR12D3*, OR12D2*, OR11A1*, OR10C1*,	
			OR2H1*, MAS1L*, OR14J1, LOC100507362, GABBR1,	
			*UBD, OR2H2*	None
8	58090000	−0.124	LOC100507651*, LOC286177*, BC048118, IMPAD1	None
10	58420000	−0.123	Intergenic	None
5	97180000	−0.123	Intergenic	None
10	74920000	−0.120	FAM149B1*, DNAJC9*, TTC18*, ECD*	6 scans
8	111770000	−0.118	Intergenic	3 scans
17	44060000	−0.114	MAPT*, CRHR1, KANSL1	None
11	38250000	−0.114	Intergenic	7 scans
7	78890000	−0.112	MAGI2*	None
4	116380000	−0.111	Intergenic	None
3	100440000	−0.110	GPR128*, TMEM45A, TFG	None
5	147530000	−0.109	SPINK5*, SPINK14*, SPINK6*, SPINK13, SPINK7, SPINK9	None
10	59720000	−0.108	Intergenic	6 scans
9	31550000	−0.108	Intergenic	None
5	26530000	−0.108	Intergenic	None
4	143970000	−0.106	*USP38*	3 scans
15	48560000	−0.106	SLC21A1*, CTXN2, DUT, FBN1,	
			*SLC24A5, MYEF2*	6 scans
17	53970000	−0.105	*PCTP, TMEM100*	None
12	59280000	−0.104	LRIG3*	None
8	30060000	−0.103	DCTN6*, TMEM66, MBOAT4, RBPMS	None
4	86090000	−0.102	*WDFY3-AS2*	None
1	238210000	−0.101	*ZP4*	None
5	145020000	−0.101	*PRELID2*	None
2	101050000	−0.101	*CHST10, NMS*	None
6	30040000	−0.101	ZNRD1*, TRIM31*, TRIM40, TRIM26, HCG17,	
			*HLA-L, HLA-J, HLA-A, HCG4B,*	
			*HLA-H, HLA-G*	None
19	22840000	−0.101	ZNF492*, ZNF99	None
2	83370000	−0.100	Intergenic	3 scans
4	133860000	−0.100	*BC040219*	None
1	66140000	−0.100	*LEPR, PDE4B*	3 scans
4	167220000	−0.100	*TLL1*	None
5	127950000	−0.100	FBN2*	None
4	35530000	−0.100	Intergenic	None

Note.—Genes marked with asterisks are coincident with the most extreme value of the statistic.

Several genes coincident with one of the 233 extreme $ksk_{\left(20\right)}^{2}$ clusters have previously been associated with functional microsatellite polymorphism. Allele size of a CA repeat in the first intron of *EGFR* ($ksk_{\left(20\right)}^{2}=-0.077$) is well known to regulate the expression of epidermal growth factor receptor, which is overexpressed in multiple tumor types and associated with asthma risk (Gebhardt et al. 1999, 2000; Wang et al. 2006; Baranovskaya et al. 2009). Kalcheva et al. (1999) found that seven copies of a CAG repeat in the 5'-UTR of *MAP2* ($ksk_{\left(20\right)}^{2}=-0.075$) were potentially protective against certain forms of dementia and stroke. Devon et al. (2001) identified a microsatellite in *GRM5* ($ksk_{\left(20\right)}^{2}=-0.096$) that may regulate the expression of this gene, which is believed to have a role in the pathology of schizophrenia (Matosin and Newell 2013). The GT repeat in *SEMA6D* ($ksk_{\left(20\right)}^{2}=-0.095$) was one of 22 dinucleotide repeats identified by Riley and Krieger (2009), whose flanking-sequence conservation suggests importance in mammalian evolution.

**Fig. 6.—:**
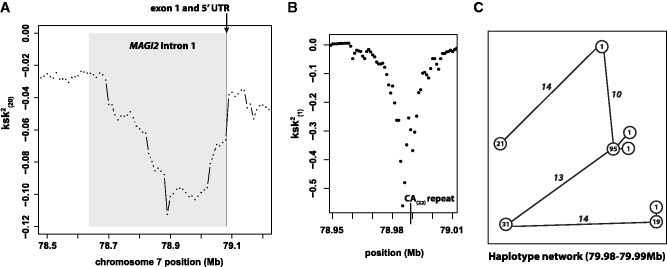
Dissecting the cluster of extreme $ksk_{\left(20\right)}^{2}$ values in intron 1 of *MAGI2*. (*A*) $ksk_{\left(20\right)}^{2}$ values in the region of chromosome 7. (*B*) High-resolution scan of a portion of the region in (*A*), where a dramatic decrease in $ksk_{\left(1\right)}^{2}$ coincides with a perfect CA repeat of length 22; each point is for a 10-kb window stepping forward 1 kb at a time. (*C*) The haplotype network of the 10-kb window with the most extreme value of $ksk_{\left(20\right)}^{2}$ in (*B*). Numbers in nodes are the number of chromosomes bearing a haplotype (out of 170), whereas numbers along vertices are the number of differences between a pair of connected haplotypes.

Troughs in the value of $ksk_{\left(20\right)}^{2}$ are driven by clusters of low $ksk_{\left(1\right)}^{2}$ values. $ksk_{\left(20\right)}^{2}$ is therefore useful for detecting anomalous areas of the genome; however, a high-resolution scan of $ksk_{\left(1\right)}^{2}$ values can be used to subsequently narrow the interval of interest. As an example, we dissected a particularly strong $ksk_{\left(20\right)}^{2}$ signal in the first intron of *MAGI2* on chromosome 7, where $ksk_{\left(20\right)}^{2}$ dropped to a minimum of −0.112 (fig. 6*A* and table 2). Specifically, we calculated $ksk_{\left(1\right)}^{2}$ for overlapping 10-kb windows in steps of 1 kb. An obvious trough in $ksk_{\left(1\right)}^{2}$ values was observed between 78.97 and 79.00 Mb, which coincided with a perfect ${\text{CA}}_{\left(22\right)}$ repeat (fig. 6*B*). The 10-kb window associated with the lowest value of $ksk_{\left(1\right)}^{2}=-0.563$ spanned between 78.98 and 78.99 Mb, where *K* = 8 and *S* = 44. The network of eight haplotypes consisted of four common haplotypes separated by large distances (fig. 6*C*).

## Discussion

### Challenges in Detecting Selection on Mutationally Complex Loci

The genome of a species comprises numerous types of genetic variants with a variety of mutational mechanisms and rates. Because of their simplicity and abundance, SNVs receive the most empirical and theoretical attention. As a result, methods used to detect selection were specifically developed to detect anomalies in sequence data that are expected when selection targets an SNV. Whether or not selection on variants with different mutational properties will produce similar effects on sequence variation is unclear.

The standard selective sweep model assumes the following: 1) At fixation, all copies of the favored variant are identical by descent and 2) the favored variant begins as a new mutation (Maynard Smith 1976). When these assumptions hold, selection is comparatively easy to detect because the selected variant is tagged by its original haplotypic background, which rises in frequency with the selected variant and generates a concomitant crash in sequence diversity.

#### Frequent Recurrent and Back Mutation

On the contrary, if a selected locus experiences common recurrent and back mutation in violation of the ISM, all copies of the favored variant need not be identical by descent. For example, many copies of the most fit allele at a microsatellite locus targeted by selection may be recent products of mutation from less fit alleles rather than direct descendants of the first chromosome to carry the favored allele size. Thus, a favored microsatellite allele may exist on several different haplotypic backgrounds, making it more difficult to detect the presence of selection using statistics that rely on substantial deformations of the SFS. The negative correlation between the prevalence of recurrent mutation and power to detect selection is demonstrated by our results for *D* and *E*, which provide very low power to detect microsatellite selection when mutation rate is high (solid lines, fig. 2*B* and *F*).

As a consequence of recurrent mutation, microsatellite selection often fails to drive a single haplotype to high frequency (fig. 4). In contrast, a single haplotype is driven to near fixation by a hard sweep targeting an SNV (fig. 4) and minor haplotypes are all highly similar to the most frequent haplotype. Indeed, most minor haplotypes at the site of a hard sweep differ from the majority haplotype at only a single site for hundreds of generations following fixation of the beneficial SNV (supplementary fig. S8, Supplementary Material online). Thus, the remaining haplotypes after a hard sweep primarily differ from each other due to recent point mutation. On the contrary, we infer that most differences between haplotypes at mutation–selection equilibrium in the microsatellite case reflect the deeply divergent ancestries of the haplotypes. These differences help explain why haplotype-based statistics provide more power than SFS-based statistics to detect microsatellite selection. Although haplotype diversity is substantially reduced by selection on a microsatellite (i.e., H and K go down), effects on linked sequence diversity across the SFS are muted by the divergent ancestries/sequences of the surviving haplotypes. Pennings and Hermisson (2006b) obtained qualitatively similar results in their investigation of SNV-based soft sweeps with recurrent mutation. However, the magnitude of effect on *K* and *S* in the case of microsatellite selection is magnified due to the frequency of mutation at a microsatellite locus. Pennings and Hermisson (2006b) considered a case where recurrent mutation was rare during the course of a selective event, back mutation was not allowed, and only two allelic states were permitted. The frequency of recurrent and back mutation at the selected microsatellite loci in our simulations provides substantially greater probability for favored allele sizes to be linked with numerous haplotypic backgrounds. As a result, $ksk_{\left(20\right)}^{2}$ is frequently driven strongly negative by microsatellite selection but not soft sweeps targeting SNVs.

#### Selection from Standing Variation

Recurrent mutation leads to association of the selected variant with multiple divergent haplotypes during the course of a selective event. As we have seen, haplotype-based statistics and $ksk_{\left(20\right)}^{2}$ can provide decent power to detect the haplotype configuration that results from this scenario. However, several authors have posited that microsatellites represent important targets of selection because high mutation rate allows these loci to accumulate extensive variation that can be drawn upon immediately when environmental conditions change (Kashi et al. 1997; King et al. 1997; Trifonov 2004). To the extent that this is true, microsatellite selection will be difficult to detect using linked sequence data. Selection on standing variation describes a situation in which the to-be selected microsatellite allele is initially (nearly) neutral. As a result, it rises in frequency embedded within a variety of haplotypes. Once selection begins, this diversity of linked haplotypes is likely to remain, and anomalous haplotype configurations are unlikely to develop.

To quantify this argument, we used $\Delta_{\text{msat}}$, which measures the distance between the allele frequency distribution of a microsatellite when selection begins and at mutation–selection equilibrium. We previously showed that this distance is positively correlated with the duration and cost of microsatellite selection (Haasl and Payseur 2013). Here, we find that $\Delta_{\text{msat}}$ also influences the selective footprint left by microsatellites under selection. High values of $\Delta_{\text{msat}}$ (>5) nearly always correspond to cases where the favored microsatellite allele does not yet exist in the population when selection begins. In other words, these are not cases of selection on standing variation. Once the favored allele is discovered via mutation, it quickly rises in frequency; due to frequent recurrent mutation, however, the favored allele size can become linked to a small number of diverse haplotypes, resulting in anomalous haplotype configurations and significant values of $ksk_{\left(20\right)}^{2}$. Conversely, low values of $\Delta_{\text{msat}}$ nearly always indicate that the favored allele has existed in the population for some time, that is, selection on standing variation.

Indeed, it appears that the value of $\Delta_{\text{msat}}$ is a strong determinant of how easy it is to identify cases of microsatellite selection using linked sequence diversity. Low values of $\Delta_{\text{msat}}$ weaken selective footprints (supplementary fig. S2, Supplementary Material online, middle column) and vice versa (fig. 1*D* and supplementary fig. S2, Supplementary Material online, right column). The most negative values of $ksk_{\left(20\right)}^{2}$ were associated with the greatest values of $\Delta_{\text{msat}}$, whereas simulations where $\Delta_{\text{msat}}<2$ produced values of $ksk_{\left(20\right)}^{2}$ that were indistinguishable from neutral simulation results (fig. 5). Given its importance to selective dynamics and because the starting allele frequency distribution is unavailable in most empirical situations, the starting distribution of allele sizes (or its proxy, $\Delta_{\text{msat}}$) presents a troubling nuisance parameter for inference of microsatellite selection. Furthermore, the larger variance observed in summary statistics for microsatellites (fig. 1) may at least be partially explained by variance in the simulated value of $\Delta_{\text{msat}}$.

### Prospects for Detecting Microsatellite Selection from Scans of Linked Diversity

Given the confounding influences of recurrent mutation and selection on standing variation, the outlook for detecting microsatellite selection using patterns of linked variation may appear bleak. This concern is realized in the case of SFS-based statistics, for which statistical power to detect selection never exceeds 50% when mutation rate is high (fig. 2). Similarly, SweepFinder fails to identify any instances of microsatellite selection (fig. 5). On the other hand, haplotype-based statistics yield moderate-to-high power to detect microsatellite selection. The long-lived power of *K* to detect selection on microsatellites with high mutation rates is perhaps particularly important. This result runs counter to the other five statistics, for which microsatellites with low mutation rate are either easier to detect or yield comparable power to microsatellites with high mutation rate.

To explain the relatively high power of *K* to detect selection on high-mutation microsatellites, consider that a neutral sequence bearing low *S* is also expected to harbor a small number of haplotypes; there are simply fewer variants and therefore fewer permutations (i.e., haplotypes). Although hard sweeps on SNVs dramatically reduce *K* (fig. 1*C* and D), they also substantially reduce *S*. Thus, low *S* and low *K* conditions are characteristic of a hard sweep after fixation but are hardly unexpected under the null hypothesis of neutrality. Although microsatellite selection also reduces *K* substantially (fig. 1*C* and *D*), recurrent mutation and/or selection from standing variation frequently result in linkage between the favored microsatellite allele and several distinct haplotypes. Thus, selection on microsatellites with high mutation rates produces a combination that is unexpected under neutrality: Intermediate *S* and low *K*. The $ksk_{\left(n\right)}^{2}$ statistic proposed here is meant to capture these diagnostic patterns (see below). Importantly, population bottlenecks should decrease both *S* and *K*, leading to patterns that do not mimic those resulting from microsatellite selection. Although the empirical null distributions we generated here incorporated previously estimated demographic history for the CEU population, formal examination of the properties of $ksk_{\left(n\right)}^{2}$ are warranted—including its sensitivity to nonequilibrium demography. In particular, the variance of $ksk_{\left(20\right)}^{2}$ may increase dramatically in nonequilibrium scenarios.

### Long-Term Microsatellite Selection and Similarity to Background Selection

Fixation of a beneficial SNV terminates the transient selective phase and its associated effect on linked diversity. However, unless selection is very strong and mutation rate is low, a favored microsatellite allele does not fix (Haasl and Payseur 2013). Instead, new mutation continuously introduces less fit alleles to the population, that is, mutation–selection equilibrium is achieved rather than fixation. The constant production of less fit microsatellite alleles in a population ensures that selection continues to act at the selected locus, thereby eliminating less fit microsatellite alleles along with their linked variants. These conditions are analogous to background selection (Charlesworth et al. 1993).

It follows that continuous selection on microsatellites with high mutation rates may cause long-term reductions in linked sequence diversity. For example, nontriplet repeats in exons might cause local depressions in linked sequence diversity if mutation rate is great enough to generate substantial numbers of deleterious alleles. Moreover, higher mutation rates at a selected microsatellite will cause more frequent production of deleterious alleles and concomitant elimination of their linked diversity. This predicts that mutation rate among genic microsatellites will be negatively correlated with flanking sequence diversity.

### Nonequilibrium Demography and $ksk_{20}^{2}$

$ksk_{\left(20\right)}^{2}$ appears to retain its power to detect both microsatellite and strong SNV selection in cases of substantial demographic change (supplementary figs. S3–S6, Supplementary Material online). This statistical power results from the fact that $ksk_{\left(20\right)}^{2}$ is driven more negative than the genomic background level of $ksk_{\left(20\right)}^{2}$ in cases of equilibrium and nonequilibrium demography (supplementary fig. S7, Supplementary Material online). Importantly, this means that real instances of microsatellite and SNV selection should be identifiable even when it is not possible to accurately estimate the demography of a population using putatively neutral loci. We also note that standardized iHS was able to detect several instances of microsatellite selection under the exponential decline scenario (supplementary fig. S3, Supplementary Material online). It is unclear why this particular combination of selective target and demographic change enables iHS to detect selection. SweepFinder identified several significant windows of composite LR for most cases of SNV and microsatellite selection under both demographic scenarios. However, significant windows were seldom contiguous as they were in the case of constant population size (fig. 5). Thus, only $ksk_{\left(20\right)}^{2}$ produced easily interpretable and significant troughs for SNV and microsatellite selection that were similar in appearance for both constant population size and the modeled instances of demographic change.

### A Scan for Nonneutral Microsatellites

Our simulations indicate that sequences demonstrating low *K* and high *S* may be predictive of microsatellite selection; the proposed statistic $ksk_{\left(n\right)}^{2}$ is sensitive to this joint condition. Moreover, after mutation–selection balance is achieved at a selected microsatellite, the popular haplotype-based statistic iHS fails to detect microsatellite selection (fig. 5). Given that selection on microsatellites may continue long after mutation–selection balance is reached (see next section), the latter finding is particularly important. However, we also found that hard sweeps with large selection coefficients deflect $ksk_{\left(n\right)}^{2}$ strongly (table 2 and fig. 5); weaker hard sweeps have no effect on $ksk_{\left(20\right)}^{2}$ (table 2). Thus, in scans of empirical genomic data, significant values of the $ksk_{\left(n\right)}^{2}$ statistic may indicate: 1) Selection on a microsatellite or 2) a strong selective event targeting an SNV. It would be preferable to identify a statistic that only detected microsatellite selection. However, consider that iHS returns significant hits when the target of selection is a microsatellite that has not reached mutation–selection balance; it is not specific to SNV selection.

These considerations are particularly important to the interpretation of our scan of the autosomes using $ksk_{\left(20\right)}^{2}$. The 233 clusters of significant $ksk_{\left(20\right)}^{2}$ values across the autosomes include (or are within 1.5 Mb of) the most commonly reported targets of natural selection in European populations (table 2 and supplementary table S1, Supplementary Material online; see Results). Given that most of these targets are known to be SNVs, these results confirm the ability of a $ksk_{\left(20\right)}^{2}$ scan to detect SNV targets of strong selective sweeps in particular.

Yet, the results of our genomic scan also suggest that $ksk_{\left(20\right)}^{2}$ detects novel targets of selection, many of which are likely to be microsatellites. Twenty-seven of the top 37 clusters of significant $ksk_{\left(20\right)}^{2}$ values coincide with genomic regions that have not been identified by previous genome-wide scans for selection (table 2). This fact could, of course, simply point to the identification of spurious targets of selection. However, our dissection of the trough in $ksk_{\left(20\right)}^{2}$ coincident with intron 1 of *MAGI2* suggests that the novel regions we identified are in fact plausible candidates for selection targets. Using overlapping windows of $ksk_{\left(1\right)}^{2}$, we localized the strongest signal of low *K* and high *S* to a 10-kb window that includes a perfect CA repeat of length 22 in the human reference sequence (fig. 6*B*). Furthermore, the haplotype configuration of this 10-kb window (fig. 6*C*) is in striking agreement with simulations of strong microsatellite selection and high mutation rate: Most common haplotype at 50%, second most common at 20%, and third most common at 10% (cf. fig.4, ϕ=5,g=−0.05). Indeed, this is the proposed reason for the effectiveness of $ksk_{\left(20\right)}^{2}$. Strong microsatellite selection coupled with high mutation rate drives a small number of highly distinct haplotypes to high frequencies (i.e., low *K* and high *S*).

It is interesting that 15 of the 233 clusters of significant $ksk_{\left(20\right)}^{2}$ values coincide with gene duplication clusters, such as those of zinc finger and olfactory receptor genes (see Results). Given that likely targets of CNV (copy number variation) selection such as *DPP10* (Girirajan et al. 2013) were also detected by our $ksk_{\left(20\right)}^{2}$ scan, it seems possible that $ksk_{\left(20\right)}^{2}$ possesses capacity to identify a variety of multiallelic targets of selection.

Three factors other than selection that commonly affect haplotype configuration are demographic change, variation in recombination rate, and sampling error. These factors must be considered as alternative explanations for the patterns observed. Demography seems an unlikely explanation, as our empirical null distribution incorporated a recent estimate of the demographic history of the CEU population (supplementary material, Supplementary Material online; Gravel et al. 2011). Low rates of recombination provide an alternative neutral explanation for low values of $ksk_{\left(20\right)}^{2}$. Therefore, it is important to compare the recombination rate of any region of interest with the genome-wide distribution of recombination rates. However, based on a recent high-resolution estimate of human recombination rates (Kong et al. 2010), only one of the top 37 clusters of significant $ksk_{\left(20\right)}^{2}$ values possesses an unusually low recombination rate compared with the genomic average. The converse problem is that locally high recombination rates may obscure anomalous values of $ksk_{\left(20\right)}^{2}$. Indeed, observed values of $ksk_{\left(20\right)}^{2}$ tend to bow upward near the telomeres, which are generally associated with higher local recombination rates. This suggests that a genome-wide level of significance for $ksk_{\left(20\right)}^{2}$ as used here is conservative, as it may result in false negatives near telomeres or recombination hotspots. Finally, we note that 1000 Genomes data used here are based on very low sequence coverage genomes. Localized sampling error caused by particularly low coverage in a region might therefore explain some of the anomalous regions. However, none of the top 37 clusters of $ksk_{\left(20\right)}^{2}$ values were associated with low coverage regions in the 1000 Genomes data, including that of the promising *MAGI2* locus.

### Uncertainty Regarding Selective Regime and Strength

Although empirical results suggest that additive or multiplicative models are the most biologically plausible forms of microsatellite selection (Vinces et al. 2009; Gemayel et al. 2010), the frequency and dynamics of microsatellite selection are not truly known. We emphasize that different selective regimes may produce selective footprints far different from those suggested by the results of our simulations. In particular, any selective regime that causes the identity of the most fit allele size to change over time may affect patterns of linked variation differently. The patterns generated by microsatellite selection here rely on the fact that there is a target allele size toward which the allele frequency distribution progresses. However, the most plausible targets of positive microsatellite selection seem to be those that cause changes in gene expression (Rockman and Wray 2002; Trifonov 2004; Vinces et al. 2009). And, in these cases, a specific “best” allele size is targeted. Ultimately, without more definitive empirical guidance, it is difficult to be more specific with our models of selection. It is also difficult to equalize selective strength between the scenarios of microsatellite and SNV-based selection. The parameters used to impose selection—*s* for SNVs and *g* for microsatellite selection—have different interpretations. Thus, there is some concern that differences between the power of the statistics observed in our simulations of SNV and microsatellite selection may reflect differences in simulated selective strength rather than divergent mutational mechanisms. However, we note that mutation had a greater influence on the power of different statistics to detect microsatellite selection than the choice of selection parameter *g*, for example, in figure 2*A*, *B*, and D, dashed lines (low mutation, high and low values of *g*) are more similar to one another than solid lines (high mutation, high and low values of *g*). The same is true of haplotype configuration (fig. 4). This suggests that mutational dynamics have a greater influence on the selective footprint left by microsatellite selection than the value of the selection parameter, minimizing the effect of possible disparities between selective strength in the SNV and microsatellite cases.

### Implications

As evolutionary geneticists scan the genomes of greater numbers of species and populations, it is incumbent upon us to consider the varied ways in which genomes might record instances of natural selection. Studies of the effects of natural selection on linked sequence diversity have largely overlooked the consequences of complex mutation. Our goal was to determine whether this complicating factor modifies the standard expectations of how selection affects linked diversity. Moreover, we hoped to identify a means for detecting selection targeting microsatellites, the best studied class of genetic variant that exhibits complex mutation.

All six of the sequence summary statistics tested here possess some power to detect microsatellite selection as modeled (figs. 2 and 3). This indicates that under certain conditions, microsatellite selection does affect linked sequence diversity in a manner comparable with that of selection on SNVs. An important implication of this result is that we should not assume that significant values of, for example, Tajima’s *D* result from selection on an SNV. On the other hand, SFS-based statistics bear substantially less power to detect selection on microsatellites, particularly when the mutation rate is high (fig. 2). This implies that scans for selection using only the most common scanning statistics have considerable potential to miss evidence of important instances of natural selection. Thus, the proposed statistic may be useful in identifying noncanonical effects of natural selection on linked sequence diversity, and, thereby, non-SNV targets of selection.

## Simulation Program

The simulation software written to perform the simulations in this article is available for download and installation from http://www.uwplatt.edu/biology/ryan-haasl/ (last accessed June 29, 2014).

## Supplementary Material

Supplementary figures S1–S8, table S1, and text are available at *Genome Biology and Evolution* online (http://www.gbe.oxfordjournals.org/).

Supplementary Data
