# Supplementary material for: The Effects of Microsatellite Selection on Linked Sequence Diversity
Source: Genome Biol Evol. 2014 Jun 19;6(7):1843–61. doi: 10.1093/gbe/evu134 (PMC4122932; doi:10.1093/gbe/evu134)
Supplement: Supplementary Data [file supp_6_7_1843__index.html]

The effects of microsatellite selection on linked sequence diversity — The Effects of Microsatellite Selection on Linked Sequence Diversity — Supplementary Data 

# The Effects of Microsatellite Selection on Linked Sequence Diversity

## Supplementary Data

files

**Files in this Data Supplement:**

- Supplementary Data - zip file
