# Supplementary material for: The Effects of Microsatellite Selection on Linked Sequence Diversity
Source: Genome Biol Evol. 2014 Jun 19;6(7):1843–61. doi: 10.1093/gbe/evu134 (PMC4122932; doi:10.1093/gbe/evu134)

Supplementary Material

Generating the empirical distribution of $ksk^2_{(20)}$ for the CEU population

We used the demographic model estimated by Gravel et al. (2011) to guide the selection of prior distributions for demographic parameters related to the history of the CEU population. Under this model an African population of size N_A undergoes an instantaneous bottleneck at time T_B to size N_B. At time T_C, which represents the split between European and Asian lineages, another bottleneck occurs, reducing population size to N_{CEU}. Immediately following this decrease, exponential population growth begins, eventually resulting in a current population size of N_0. Based on the results of Gravel et al. (2011), we used the following uniform priors:

- N_A: 13,419-16,184 individuals
- T_B: 45-69 kya
- N_B: 1,453-2,494 individuals
- T_C: 21-27 kya
- N_{CEU}: 677-1290 individuals
- α, exponential growth rate from T_C to present: 0.0029-0.0052

In MS, all population size changes and event times are in terms of the current population size N_0. Thus, for each simulation we drew parameters values in terms of the units listed above and used. Then we used α and the randomly drawn value of N_{CEU} to calculate the corresponding value of N_0, after which we scaled all size and time parameters for use in MS. We assumed a point-mutation rate of $\mu = 1\text{e-}08$ per site. For recombination rate, we drew from the uniform prior 1e-10 - 2e-08. Each simulation generated a 2Mb sequence. Although there was inter-simulation variability in recombination rate, individuals simulations used a constant recombination rate.

The following MS command was used for each simulation. Actual values of the parameters in angled brackets depended on draws from prior distributions. $\theta = 4N_0\mu$.

./ms 170 1 -t $\langle\theta\rangle$ -r $\langle\rho\rangle$ 2000000 -G $\langle\alpha\rangle$ -eN $\langle T_C\rangle$ $\langle N_B/N_0\rangle$ -eN $\langle T_B\rangle$ $\langle N_A/N_0\rangle$

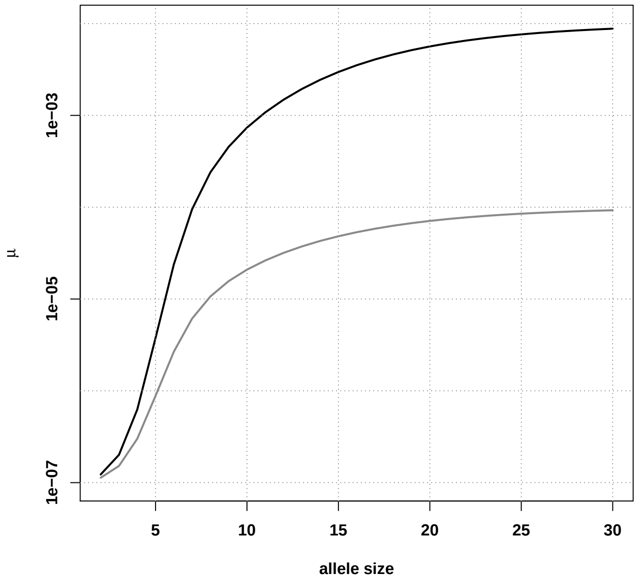

Figure S1: Mutation curves simulated in this study. Our model of allele-specific mutation rate requires specification of three parameters: ψ controls the allele size at which mutation rate begins to increase, ϕ controls the maximum mutation rate, and γ controls the slope of increase. In all simulations, we used $\psi = 2$ and $\gamma = 0.15$ while ϕ was either set to 3 or 5. In a few instances, we set $\phi = 4$. The lower curve corresponds to $\phi = 3$, while the upper curve corresponds to $\phi = 5$.

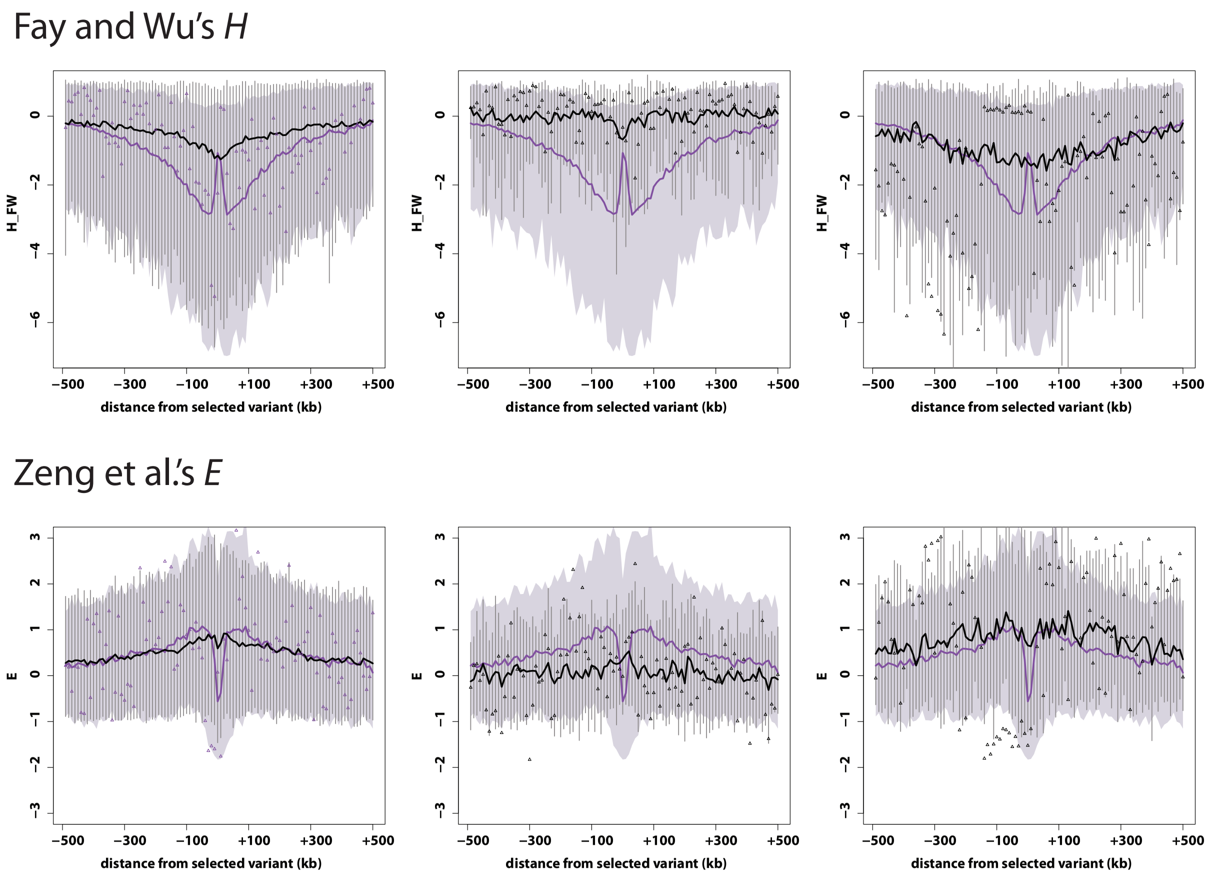

Figure S2: The spatial footprint of hard sweeps and microsatellite selection measured by Fay and Wu's H_{FW} (top row) and Zeng et al.'s E (bottom row). The first panel of each row summarizes each statistic across 500 simulation replicates of a hard sweep (s = 0:05, h = 0.5) or microsatellite selection (ϕ = 5,g = -0.05). Lines show the mean value of each statistic for hard sweeps (black) or microsatellite selection (purple). The 5%-95% interquantile range is shown as a light purple cloud (hard sweeps) or vertical bars (microsatellite selection). The middle column only includes replicates of microsatellite selection where Δ_{msat} was among lowest 10% of those recorded. The right hand column only includes replicates of microsatellite selection where Δ_{msat} was among the highest 10% of those recorded. All panels also show results for a single, representative replicate, where the value of the statistic for each windows is represented by a triangle (purple=hard sweep; black=microsatellite selection)

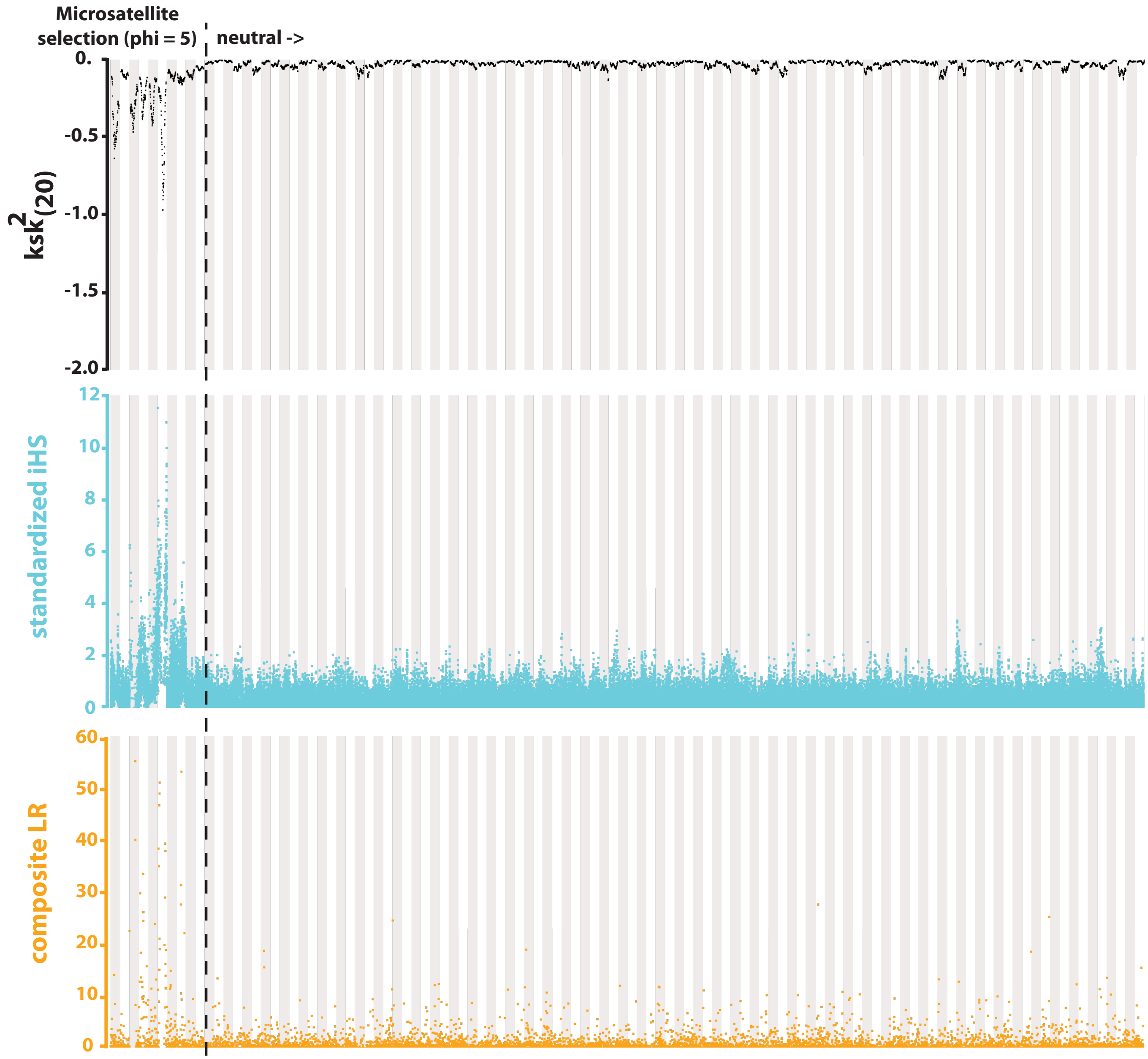

Figure S3: Comparison of $ksk^2_{(20)}$, standardized iHS, and the composite likelihood ration (LR) for neutral and **microsatellite selection** ($\phi = 5$; $g = 5$) simulations under the **exponential decline demographic scenario**. Results from 110 simulations of a 1Mb sequence are shown. In cases of selection, the selected microsatellite is position at the exact center of the 1Mb sequence. The 10 simulations of selection are to the left of the dotted line and 100 neutral simulations are to the right of the dotted line. Separate simulations are indicated by alternating background color.

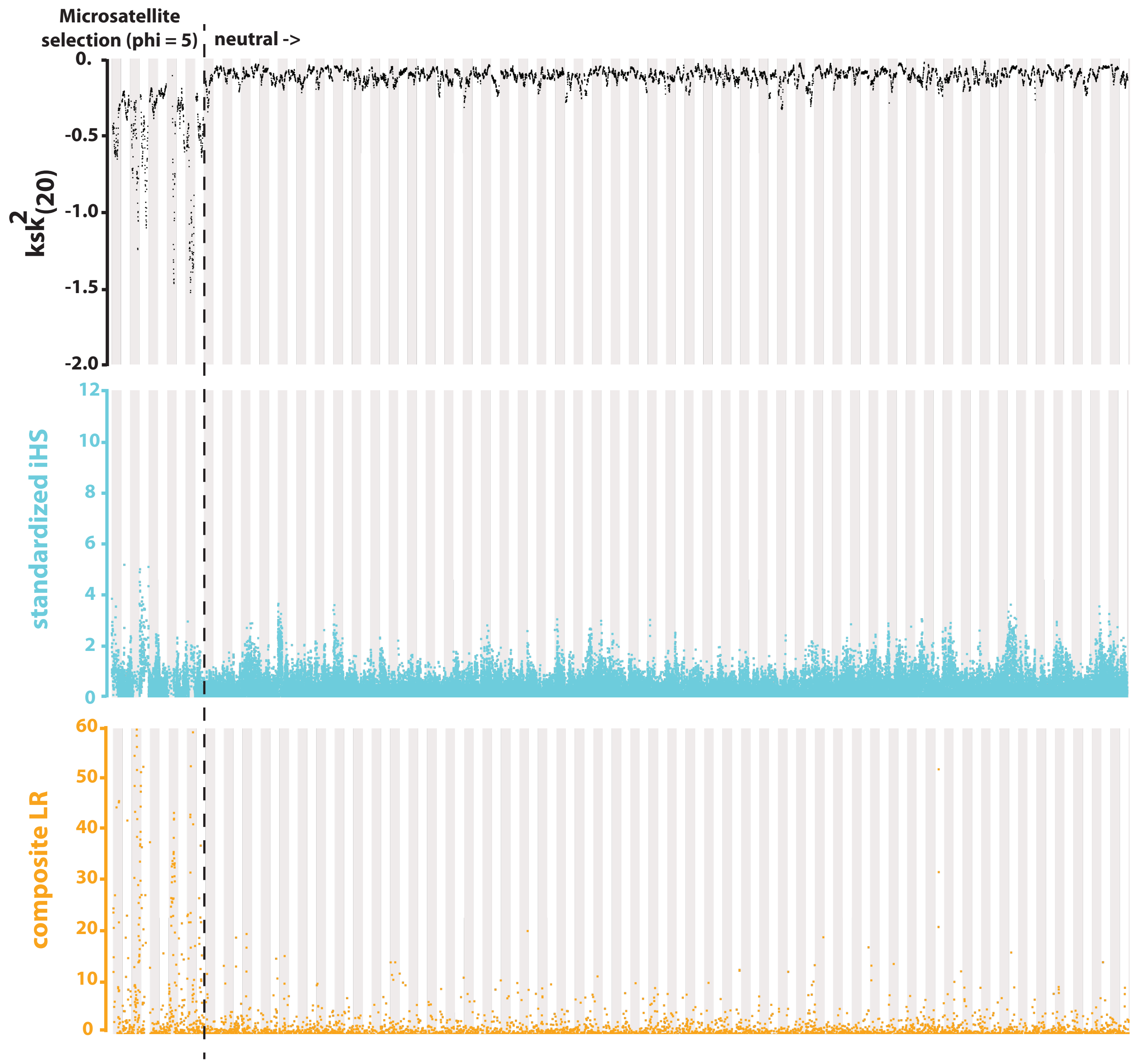

Figure S4: Comparison of $ksk^2_{(20)}$, standardized iHS, and the composite likelihood ration (LR) for neutral and **microsatellite selection** ($\phi = 5$; $g = 5$) simulations under the **bottleneck-expansion demographic scenario**. Results from 110 simulations of a 1Mb sequence are shown. In cases of selection, the selected microsatellite is position at the exact center of the 1Mb sequence. The 10 simulations of selection are to the left of the dotted line and 100 neutral simulations are to the right of the dotted line. Separate simulations are indicated by alternating background color.

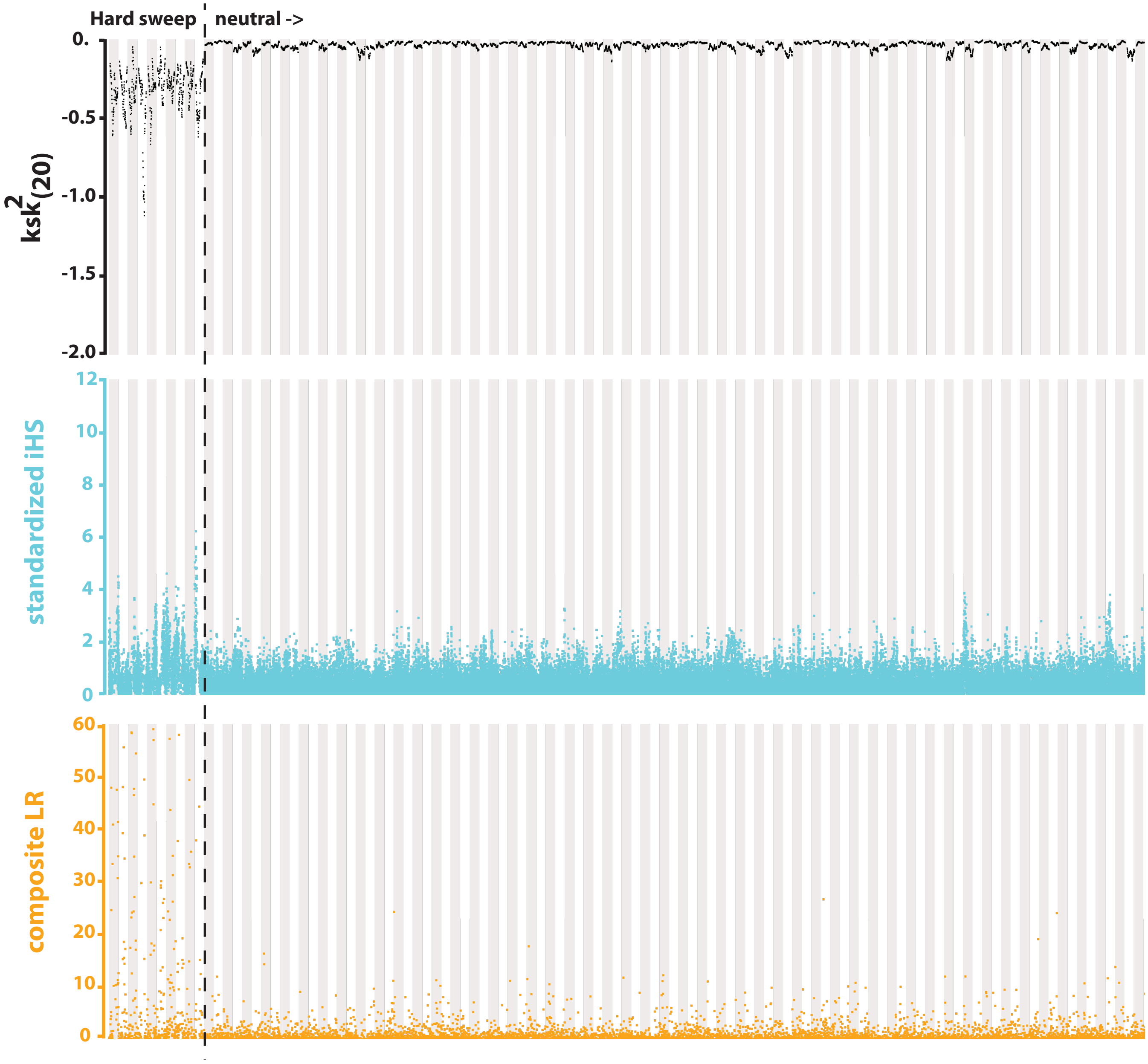

Figure S5: Comparison of $ksk^2_{(20)}$, standardized iHS, and the composite likelihood ration (LR) for neutral and **SNV selection ($s = 0.05$, hard sweep)** simulations under the **exponential decline demographic scenario**. Results from 110 simulations of a 1Mb sequence are shown. In cases of selection, the selected microsatellite is position at the exact center of the 1Mb sequence. The 10 simulations of selection are to the left of the dotted line and 100 neutral simulations are to the right of the dotted line. Separate simulations are indicated by alternating background color.

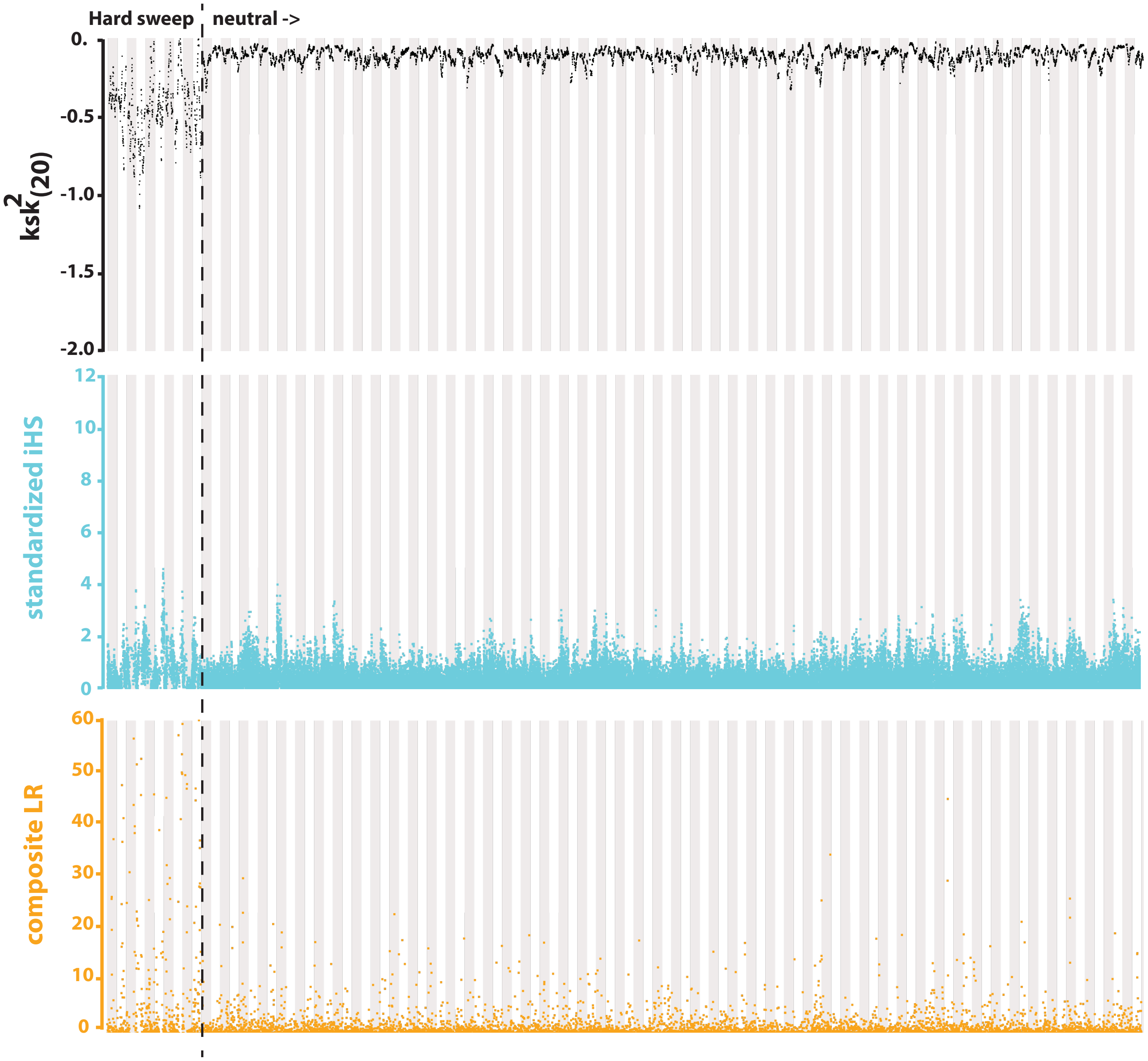

Figure S6: Comparison of $ksk^2_{(20)}$, standardized iHS, and the composite likelihood ration (LR) for neutral and **SNV selection (**s **= 0.05, hard sweep)** simulations under the **bottleneck-expansion demographic scenario**. Results from 110 simulations of a 1Mb sequence are shown. In cases of selection, the selected microsatellite is position at the exact center of the 1Mb sequence. The 10 simulations of selection are to the left of the dotted line and 100 neutral simulations are to the right of the dotted line. Separate simulations are indicated by alternating background color.

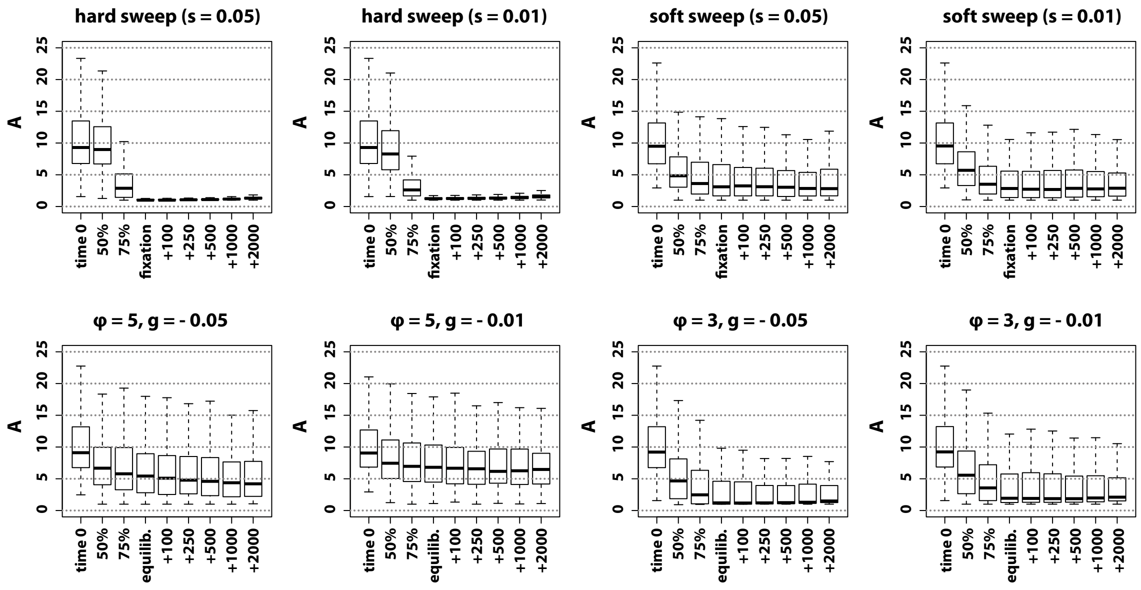

Figure S8: The statistic A quantifies the difference between the most common haplotype and all other haplotypes:

$$A = \sum_{i=2}^{K} n_i d(h_1, h_i) \ / \ \sum_{i=2}^{K} n_i$$

where h_i is the ith haplotype in the ordered (by descending frequency) set of observed haplotypes, $d(h_i, h_j)$ is the number of pairwise differences between haplotypes i and j, and n_i is the count of haplotype i. In words, A is the average number of pairwise differences between the most common haplotype and all secondary haplotypes. For samples with more than one haplotype, the lower bound on A is unity, which indicates all secondary haplotypes differ from the most common haplotype at a single site. When only one haplotype is present in the sample, A is undefined.

Table 1: Autosomal scan for natural selection, population CEU, $n = 170$ chromosomes

chromosome	position (hg19)	ksk^2_{20}	genes
1	53570000	-0.0862523924	SLC1A7*, PODN*, SCP2*, CPT2, LPR8, MAGOH, ECHDC2
1	66140000	-0.099865874	LEPR, PDE4B
1	70510000	-0.0794043399	LRRC7*, LRRC40, SRSF11
1	73140000	-0.0836355876	intergenic
1	89170000	-0.0865662283	PKN2*, GTF2B, AK123834
1	100490000	-0.0752274077	SLC35A3*, HIAT1*, SASS6*, TRMT13, DBT, AGL
1	102150000	-0.087878387	OLFM3
1	102770000	-0.0785323259	intergenic
1	104630000	-0.0832619976	LOC100129138
1	118410000	-0.083527367	GDAP2*, WDR3, SPAG17
1	183660000	-0.0802634277	RGL1*, ARPC5*, APOBEC4*, NCF2, SMG7
1	188830000	-0.0938879173	intergenic
1	189870000	-0.0853381867	FAM5
1	215020000	-0.0770741287	KCNK2, CENPF
1	221750000	-0.0860223981	DUSP10
1	238210000	-0.1017698717	ZP4
2	7700000	-0.075549537	LOC100506274
2	13580000	-0.0939253038	intergenic
2	17230000	-0.0902946686	intergenic
2	21890000	-0.0782485629	LOC645949
2	83370000	-0.1002094442	intergenic
2	101050000	-0.1010192644	CHST10, NMS
2	116270000	-0.0875156241	DPP10*
2	117230000	-0.0824681594	intergenic
2	135480000	-0.0885809846	TMEM163*, ACMSD*, LOC100129961, CCNT2
2	136070000	-0.0975136935	ZRANB3* (LCT less than 500kb away)
2	137180000	-0.0851920945	intergenic
2	137750000	-0.0893907439	THSD7B*
2	138560000	-0.0910225027	THSD7B, HNMT
2	143450000	-0.0943337335	KYNU
2	157770000	-0.0790950547	intergenic
2	163640000	-0.0980971415	KCNH7*
2	168020000	-0.0862131931	XIRP2*
2	193200000	-0.0855269362	TMEFF2
2	210280000	-0.0753023573	MAP2*
2	218560000	-0.07755703	DIRC3*, TNS1
3	17500000	-0.0870171454	TBC1D5*
3	29680000	-0.0913513422	RBMS3*
3	95740000	-0.0928541028	intergenic
3	98020000	-0.0809353269	OR5H6*, OR5H15*, OR5H1, OR5H14, OR5K4, OR5K3, OR5K1, OR5K2
3	100440000	-0.1095285711	GPR128*, TMEM45A, TFG

3	102740000	-0.0778530793	intergenic
3	110270000	-0.0903560907	intergenic
3	111050000	-0.0784439774	PVRL3
3	112270000	-0.0764582731	BTLA*, ATG3*, SLC35A5*, CCDC80*, CD200
3	113140000	-0.0852041051	WDR52*, SPICE1*, SIDT1, BOC
3	131360000	-0.0794516797	CPNE4*, MRPL3
3	144210000	-0.0810603132	intergenic
3	147920000	-0.0792241367	intergenic
3	154270000	-0.0859520158	GPR149
3	161140000	-0.093199528	NMD3, SPTSSB, LOC101243545*, OTOL1
3	162690000	-0.0862802482	BC073807*
3	165930000	-0.0903006285	intergenic
3	171420000	-0.0754634692	PLD1*, TMEM212
3	172570000	-0.0834949034	SPATA16*, ECT2*, NCEH1
4	5340000	-0.0796049103	STK32B*, C4orf6
4	12610000	-0.0834911503	intergenic
4	13410000	-0.0793404551	RAB28*, LOC285547, NKX3-2, BOD1L1
4	27590000	-0.0770322389	intergenic
4	32280000	-0.0884979625	intergenic
4	33280000	-0.0876857248	intergenic
4	34390000	-0.0864837581	intergenic
4	35530000	-0.0996542392	intergenic
4	38800000	-0.0795313694	TLR10*, TLR1*, TLR6*, FAM114A1, TMEM156, KLF3, FLJ13197
4	56090000	-0.0955328688	KDR, SRD5A3
4	64610000	-0.1343968719	intergenic (ksk1=-1)
4	69670000	-0.0787438001	UGT2B10*, UGT2B15, UGT2A3
4	71640000	-0.0811459965	RUFY3*, GRSF1*, MOB1B, IGJ, ENAM, AMBN
4	72850000	-0.0769436897	NPFFR2*, GC
4	78280000	-0.0825094782	CCNG2, CXCL13
4	79900000	-0.0779132805	BMP2K*, PAQR3*, LOC100505875*,
4	80690000	-0.0851763162	PCAT4, ANTXR2
4	82020000	-0.0750211748	BMP3*, PRKG2*, C4orf22
4	84980000	-0.0819285049	BC005018*, AK095285*
4	86090000	-0.1023921765	WDFY3-AS2
4	87630000	-0.0759557219	PTPN13*, MAPK10*, LSC10A6, C4orf36
4	96620000	-0.0755453985	UNC5C, PDHA2
4	101740000	-0.0851333689	EMCN-IT3
4	115480000	-0.0776675764	UGT8
4	116380000	-0.1105409369	intergenic
4	117170000	-0.0791257532	intergenic
4	124110000	-0.0981217634	SPATA5* (FGF2 very near by)
4	131390000	-0.0829340241	intergenic
4	133860000	-0.0999894535	BC040219
4	137510000	-0.0871100656	intergenic
4	143970000	-0.1063115694	USP38
4	148680000	-0.1385627919	PRMT10*, TMEM184C, ARHGAP10 (ksk1=-2)

4	167220000	-0.0997989007	TLL1
4	171510000	-0.1414030566	intergenic
4	172020000	-0.0853160351	LOC100506122
4	173610000	-0.0848331639	GALNTL6*
4	176200000	-0.0847947425	intergenic
5	24180000	-0.0837349427	AX747383
5	26530000	-0.1075960659	intergenic
5	34540000	-0.0754396002	RAI14
5	35440000	-0.0762708956	SPEF2
5	72010000	-0.0774576164	TNPO1, AX746492
5	97180000	-0.1231134748	intergenic
5	100960000	-0.0861846325	intergenic
5	102090000	-0.0895981388	PAM*
5	121130000	-0.0819417971	FTMT*, SRFBP1
5	127950000	-0.0997662653	FBN2*
5	145020000	-0.1014115431	PRELID2
5	147530000	-0.1090276181	SPINK5*, SPINK14*, SPINK6*, SPINK13, SPINK7, SPINK9
5	150280000	-0.0968737543	ZNF300*, ZNF300P1*, IRGM*, DCTN4, SMIM3, GPX3, TNIP1
5	151770000	-0.0955133177	NMUR2*, AK001582
6	4440000	-0.077187537	KU-MEL-3
6	29380000	-0.1247432081	OR5V1*, OR12D3*, OR12D2*, OR11A1*, OR10C1*, OR2H1*, MAS1L*, OR14J1, LOC100507362, GABBR1, UBD, OR2H2
6	30040000	-0.1008199925	ZNRD1*, TRIM31*, TRIM40, TRIM26, HCG17, HLA-L, HLA-J, HLA-A, HCG4B, HLA-H, HLA-G
6	48520000	-0.081641207	intergenic
6	64900000	-0.0771971167	EYS*
6	81670000	-0.0993695987	intergenic
6	97400000	-0.0777161378	KLHL32*, NDUFAF4, GPR63, MMS22L
6	103930000	-0.0815897803	integenic
6	109610000	-0.0792097058	CCDC162P*, LOC100996634*, CD164*, C6orf185*, PPIL6, SMPD2, MICAL1, ZBTB24, CEP57L1
6	110540000	-0.0792653446	WASF1*, METTL24*, CDC40*, DDO
6	121550000	-0.0976769585	C6orf170*
6	145790000	-0.0799572525	EPM2A
6	159470000	-0.0901413866	RSPH3*, TAGAP*, FNDC1, C6orf99, OSTCP1
7	34590000	-0.0797860936	NPSR-AS1*
7	42500000	-0.0846531175	intergenic
7	46190000	-0.0807083975	intergenic
7	54970000	-0.0771343734	EGFR*, SEC61G
7	55540000	-0.090314648	LANCL2*, VOPP1*
7	78890000	-0.112462345	MAGI2*
7	81190000	-0.0829710673	AY927633*, HGF
7	113170000	-0.0789468928	intergenic
7	117880000	-0.0878497489	NAA38*, ANKRD7*

7	118580000	-0.0872638292	intergenic
7	120600000	-0.078515067	ING3*, CPED1*, TSPAN12
7	133890000	-0.0877549617	LRGUK*, SLC35B4*, EXOC4
7	142660000	-0.0768122977	KEL*, TRPV5*, OR9A2*, OR6V1*, OR6W1P*, PIP, EPHB6, TCRVB
8	12490000	-0.1392978984	LOC100506990*, LOC729732*, FAM86B2, LONRF1, LOC340357 (really weird area, ksk1=-2)
8	23970000	-0.0754055466	ADAM28
8	27730000	-0.0833321451	CCDC25*, ESCO2*, PBK*, SCARA5*, SCARA3, NUGGC
8	30060000	-0.1032665533	DCTN6*, TMEM66, MBOAT4, RBPMS
8	35660000	-0.0885023816	UNC5D*
8	58090000	-0.1242142642	LOC100507651*, LOC286177*, BC048118, IMPAD1
8	60800000	-0.0786421029	intergenic
8	63010000	-0.0803132322	NKAIN3
8	79790000	-0.0786699971	IL7*, LOC101241902, ZC2HC1A
8	89480000	-0.0971760306	MMP16
8	111770000	-0.117772579	intergenic
8	127200000	-0.090938365	intergenic
9	25150000	-0.0755666777	intergenic
9	31550000	-0.1080450994	intergenic
9	32060000	-0.0765030702	intergenic
9	87090000	-0.0811407883	SLC28A3*, NTRK2
9	99570000	-0.0893381562	ZNF510*, ZNF782*, AAED1, CDC14B, LOC441454, NUTM2G, HIATL2
9	106930000	-0.0867863795	MC2*
10	8770000	-0.0796118201	intergenic
10	23940000	-0.0966861657	KIAA1217
10	26660000	-0.0839011837	GAD2*, APBB1IP*, MYO3A
10	56100000	-0.0844653517	PCDH15*
10	58420000	-0.1234174447	intergenic
10	59080000	-0.0911302976	intergenic
10	59720000	-0.1083529541	intergenic
10	66070000	-0.0923603591	intergenic
10	68230000	-0.0857007198	CTNNA3*
10	74920000	-0.1200379099	FAM149B1*, DNAJC9*, TTC18*, ECD*, NUDT13, P4HA1
10	85290000	-0.0809748024	intergenic
10	87060000	-0.0818726123	AK097624
10	111450000	-0.0781279909	XPNPEP1
10	117540000	-0.0791823742	ATRNL1*
11	19080000	-0.081453888	MRGPRX2*, ZDHHC13*, MRGPRX1, CSRP3, E2F8
11	21760000	-0.0957144888	NELL1
11	23690000	-0.0781566547	intergenic
11	24480000	-0.078217165	LUZP2*
11	25110000	-0.0794333256	LUZP2*
11	26480000	-0.0770390288	ANO3*, MUC15*
11	34920000	-0.0903063423	APIP*, PDHX*

11	36720000	-0.0897078773	C11orf74*, RAG2, RAG1, TRAF6
11	38250000	-0.1136757661	intergenic
11	39180000	-0.0896951725	intergenic
11	42250000	-0.077420059	LOC100507205
11	55770000	-0.0970859416	OR5F1*, OR5AS1*, OR10AG1, OR5I1, OR5W2, TRIM51, ORD16, OR5L2, OR5D18, OR8I2, OR8H2, OR8H3, OR8J3, OR8K5, OR5J2
11	71650000	-0.0753446132	RNF121*, LOC100133315*, LOC100129216*, IL18BP, FAM86C1, ALG1L9P, DEFB108B, NUMA1, LRTOMT, ANAPC15, FOLR
11	88450000	-0.095835717	GRM5*
11	90420000	-0.0750007345	HP11113*
11	103270000	-0.0808863112	DYNC2H1*
11	104720000	-0.0878788138	CASP12*, CASP4*, CASP5, CASP1, CARD16
11	106080000	-0.0949361718	BC034795*, AASDHPPT, KBTBD3, MSANTD4
11	124150000	-0.076885218	OR8D1*, OR8D2*, OR8G1, OR8G2, OR10D3, OLFR959, VWA5A, OR8B2, OR8B3, OR8B4, OR8B8
12	41740000	-0.0848355877	PDZRN4*
12	45800000	-0.0769299113	ANO6*
12	58720000	-0.0823430803	intergenic
12	59280000	-0.1039889444	LRIG3*, AK093124
12	63670000	-0.0786579751	AVPR1A
12	72460000	-0.0874036029	TPH2, TBC1D15, TRHDE-AS1
13	26240000	-0.0804016413	ATP8A2*
13	37960000	-0.0862504308	POSTN
13	38930000	-0.0805254109	UFM1*
13	39780000	-0.0843351867	LHFP, NHLRC3, PROSER1
13	60270000	-0.0815927721	DIAPH3*
13	64520000	-0.0929893928	AK057471, AK098560
13	78690000	-0.08316511	RNF219-AS1*, LINC00446*, AK090854, EDNRB
13	86620000	-0.0972210735	intergenic
13	87150000	-0.0880989117	intergenic
14	40470000	-0.0808636375	intergenic
14	41560000	-0.0927915108	BX248273
14	45320000	-0.0852880106	C14orf28*, KLH28*, FAM179B
14	60610000	-0.0941910059	DHRS7*, PCNXL4*, PPM1A, LRRC9
14	66740000	-0.0780574605	intergenic
14	88470000	-0.0905481359	GALC*, GPR65*, KCNK10 (bunch of DQXXXXs)
14	96380000	-0.0884566491	LINC00617*, TCL1A, C14orf132
15	28370000	-0.0861972096	HERC2*, OCA2*
15	45240000	-0.0962066228	C15orf43*, SORD, TRIM69, DUOX2, DUOXA1, DUOXA2, DUOX1
15	47830000	-0.0946248654	SEMA6D*
15	48560000	-0.1061936767	SLC12A1*, CTXN2, DUT, FBN1, SLC24A5, MYEF2
15	76240000	-0.0773311251	UBE2Q2*,FBXO22*, NRG4*, C15orf27
17	29100000	-0.0752268833	SUZ12P1*, CRFL3*, ATAD5*, TEFM, ADAP2, RNF13, LRRC37BP1

17	44060000	-0.1140873401	MAPT*, CRHR1, KANSL1
17	50890000	-0.089090693	C17orf112
17	53970000	-0.1051957342	PCTP, TMEM100
17	58920000	-0.0965670051	BCAS3*, PPM1D (ksk1=-0.75)
17	64080000	-0.0932041171	CEP112*
18	30670000	-0.0952286134	CCDC178*
18	40070000	-0.0938360216	LINC00907*
18	50290000	-0.0814177359	DCC*
18	52590000	-0.0799411998	CCDC68*, RAB27B, AK093940
18	58410000	-0.1381158453	intergenic
18	61550000	-0.0955630967	SERPINB2*, SERPINB11, SERPINB7, HMSD, SERPINB8
18	66650000	-0.0762532804	CCDC102B*
18	71880000	-0.0840562396	CYB5A*, C18orf63, TIMM21, FBX015
19	22840000	-0.1006041029	ZNF492*, ZNF99
19	40560000	-0.0857219939	ZNF780B*, ZNF780A*, ZNF546, MAP3K10, TTC9B, CNTD2, AKT2, FCGBP
20	6410000	-0.0762981339	intergenic
20	7370000	-0.0771265234	intergenic
20	24190000	-0.0920774997	FLJ33581*
20	53730000	-0.0849316564	intergenic
20	59310000	-0.0803815985	LOC284757
21	25480000	-0.0765403744	intergenic
21	32070000	-0.0817073155	KRTAP21-3*, KRTAP20-2, KRTAP20-3, KRTAP20-1, KRTAP20-4, KRTAP19-6, KRTAP19-7, KRTAP22-2, KRTAP6-2, KRTAP6-3, KRTAP22-1, KRTAP21-2, KRTAP21-1, KRTAP8-1, KRTAP7-1, KRTAP11-1

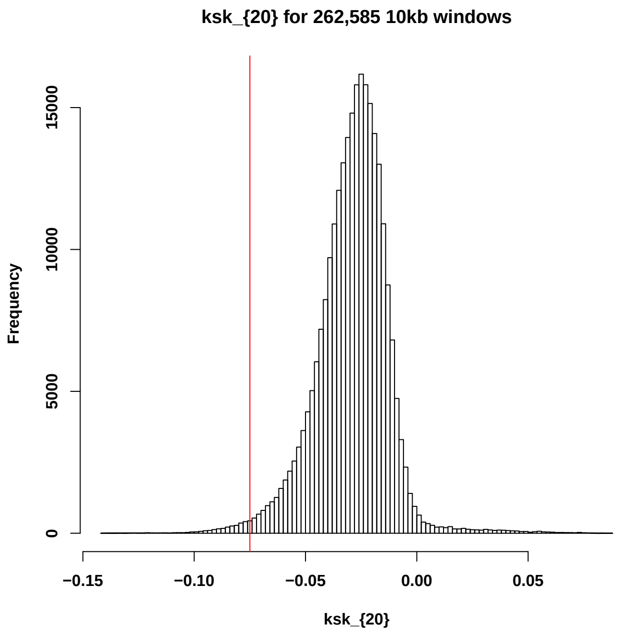

Supplement: Supplementary Data [file supp_evu134_suppl_data.zip › gbe_supplementary.pdf]
